# Temperature promotes selectivity during electrochemical CO_2_ reduction on NiO:SnO_2_ nanofibers†

**DOI:** 10.1039/d4ta04116j

**Published:** 2024-08-08

**Authors:** M. A. Rodriguez-Olguin, R. Lipin, M. Suominen, F. Ruiz-Zepeda, E. Castañeda-Morales, A. Manzo-Robledo, J. G. E. Gardeniers, C. Flox, T. Kallio, M. Vandichel, A. Susarrey-Arce

**Affiliations:** a Mesoscale Chemical Systems, MESA+ Institute, University of Twente P. O. Box 217 Enschede 7500AE The Netherlands; b Department of Chemical Engineering, MESA+ Institute, University of Twente P. O. Box 217 Enschede 7500AE The Netherlands a.susarreyarce@utwente.nl; c School of Chemical Sciences and Chemical Engineering, Bernal Institute, University of Limerick Limerick V94 T9PX Republic of Ireland matthias.vandichel@ul.ie; d Department of Chemistry and Materials Science, Aalto University School of Chemical Engineering Kemistintie 1 02015 Espoo Finland tanja.kallio@aalto.fi; e Department of Materials Chemistry, National Institute of Chemistry Hajdrihova 19 1000 Ljubljana Slovenia; f Department of Physics and Chemistry of Materials, Institute of Metals and Technology Lepi pot 11 Ljubljana Slovenia; g Instituto Politécnico Nacional, Laboratorio de Electroquímica y Corrosión, Escuela Superior de Ingeniería Química e Industrias Extractivas Av. Instituto Politécnico Nacional S/N, Unidad Profesional Adolfo López Mateos CP 07708 CDMX Mexico; h Department of Electrical Energy Storage, Iberian Centre for Research in Energy Storage, Campus University of Extremadura Avda. de las Letras, s/n 10004 Cáceres Spain cristina.flox@ciiae.org

## Abstract

Electrolyzers operate over a range of temperatures; hence, it is crucial to design electrocatalysts that do not compromise the product distribution unless temperature can promote selectivity. This work reports a synthetic approach based on electrospinning to produce NiO:SnO_2_ nanofibers (NFs) for selectively reducing CO_2_ to formate above room temperature. The NFs comprise compact but disjoined NiO and SnO_2_ nanocrystals identified with STEM. The results are attributed to the segregation of NiO and SnO_2_ confirmed with XRD. The NFs are evaluated for the CO_2_ reduction reaction (CO_2_RR) over various temperatures (25, 30, 35, and 40 °C). The highest faradaic efficiencies to formate (FE_HCOO^−^_) are reached by NiO:SnO_2_ NFs containing 50% of NiO and 50% SnO_2_ (NiOSnO50NF), and 25% of NiO and 75% SnO_2_ (NiOSnO75NF), at an electroreduction temperature of 40 °C. At 40 °C, product distribution is assessed with *in situ* differential electrochemical mass spectrometry (DEMS), recognizing methane and other species, like formate, hydrogen, and carbon monoxide, identified in an electrochemical flow cell. XPS and EELS unveiled the FE_HCOO^−^_ variations due to a synergistic effect between Ni and Sn. DFT-based calculations reveal the superior thermodynamic stability of Ni-containing SnO_2_ systems towards CO_2_RR over the pure oxide systems. Furthermore, computational surface Pourbaix diagrams showed that the presence of Ni as a surface dopant increases the reduction of the SnO_2_ surface and enables the production of formate. Our results highlight the synergy between NiO and SnO_2_, which can promote the electroreduction of CO_2_ at temperatures above room temperature.

## Abstract 2

Nanofibers composed of nickel and thin oxide nanocrystals for high-temperature electrochemical CO_2_ reduction to formate.

## Graphical abstract

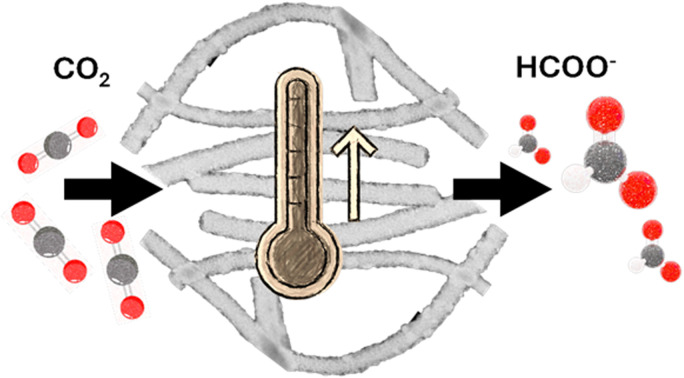

## 1. Introduction

Anthropogenic CO_2_ emissions are a problem that must be addressed to decelerate greenhouse effects like global warming. According to the Intergovernmental Panel on Climate Change (IPCC) published in 2021, the planet's temperature may increase by 1.5 °C in the following decades due to the accelerated release of CO_2_ and other greenhouse gases, posing a threat to the global ecosystems and humankind.^1^ A call-to-action part of the energy transition goals is CO_2_ recycling and utilization. One way to recycle and utilize CO_2_ is by producing CO_2_-based value-added products electrochemically. For example, electrochemical CO_2_RR can enable the production of C1 (*e.g.*, CO, HCOO^−^, CH_4_) and C2 (*e.g.*, C_2_H_4_, alcohol) products. The selectivity of C1 or C2 products is often attributed to the catalyst type or the operation conditions during CO_2_ electrolysis. As for the catalysts, three different groups of metals have been recognized based on the product generated: formate (*e.g.*, Sn,^2^ Pb,^3^ Bi,^4,5^ In,^6^ Hg^7^), CO (Au,^8^ Ag,^9^ Pd,^10^ Zn,^11^ Ni^12^), hydrocarbons and alcohols (Cu^13,14^) and other multi-carbon products like amino acids,^15^ which in combination with CO_2_ and nitrogenated sources (NH_4_HCO_3_) has been demonstrated to lead to the formation of serine.^16,17^ Among the mentioned metals, Cu is a catalyst capable of producing C1, C2, and higher multi-carbon products.^18^ However, copper exhibits low selectivity, resulting in a mixture of gaseous and liquid reaction compounds. Therefore, the selective production of CO_2_-reduction products is a key challenge that needs attention. One way to overcome this challenge is by synthetically designing a catalyst with earth-abundant elements that can cope with CO_2_RR on an industrial level aimed at 90% conversion to a single product and elevated electrolyzer temperatures for practical reasons (*e.g.*, increased reaction rates or reduced overpotentials).

The reaction temperature is a crucial yet sometimes overlooked parameter in electrochemistry that can compensate for the thermal losses during CO_2_ electrolysis. Mizuno *et al.* have delved into the electrochemical reduction of CO_2_ with various electrode compositions.^19^ In a different study, Kim *et al.* analyzed the effect of operating conditions, including temperature, on the electrochemical conversion of CO_2_ to formic acid.^20^ Like Mizuno *et al.*,^19^ the results underscore the intricate relationship between temperature and selectivity for CO_2_RR. Recently, Löwe *et al.* addressed in depth how temperature variations can influence transport and, thus, formate production.^21^ According to the authors, the temperature notably impacts the reaction selectivity at an industrially accepted current density of 200 mA cm^−2^. Specifically, the FE_HCOO^−^_ decreases from 89% at 20 °C to 85% at 70 °C, while the production of CO increases from around 7% to 11%. Interestingly, variations of about ±10% during FE_HCOO^−^_ have been observed at the assessed temperatures with current densities of up to 1000 mA cm^−2^. The results from Löwe *et al.* indicate that with an increase in temperature, the selectivity towards formate decreases while it increases for other products like CO or H_2_. Other studies reported similar trends, ascribing the decreased formate production and selectivity with increasing temperature to the kinetic effects and CO_2_ solubility.^22^ Under other conditions, *e.g.*, by varying the electrolyte solution, a fundamental understanding of the relationships between the surface coverage, pH, and kinetics has been proposed for Cu.^18,23^ The previously cited seminal works are therefore used as a stepping stone to understand the temperature effects over NiO:SnO_2_ catalyst, whose synergistic combinations can lead to tuning the selectivity of CO_2_RR products. It should be noted that other studies for NiO, SnO_2_, and their combinations do not deal with temperature variation conditions during CO_2_ electrolysis (Tables S1–S3†). NiO typically leads to CO, CH_4_, and, in some cases, to HCOO^−^ (Table S1†), while SnO_2_ leads to HCOO^−^ as the main product (Table S2†). However, their synergistic effect has only been shown HCOO^−^ products near room temperature (Table S3†).

An additional expected challenge for a single oxide composition like SnO_2_ is its instability under cathodic electrochemical potentials.^24^ The instability of SnO_2_ during CO_2_RR can be attributed to the thermodynamic formation of various oxidized tin species.^25^ Mu *et al.* highlighted the dominance of hydroxyl radicals in the reoxidation of oxide-derived metals like Cu. Despite being thermodynamically unstable under cathodic conditions, the authors propose that the presence of Cu^*δ*+^ species enables the CO_2_RR.^26^ The results from Mu *et al.* emphasize the importance of the stability of metal oxide catalysts.^26^ Lately, Jiang *et al.* have discussed the importance of stabilizing the oxidation state of SnO_2_ to achieve highly selective CO_2_ electroreduction to formate.^27^ Their study emphasizes the necessity of maintaining the oxidation state of tin during the CO_2_RR, achieved by incorporating Cu single atoms into the SnO_2_ lattice. Like single-atom catalysis, other strategies such as engineering the defects in oxides, have been proposed to tune the selectivity of CO_2_RR^28^ and NO_*x*_ reduction reactions.^28,29^ From the later examples, catalyst discovery should favor thermodynamically stable catalytic species that appropriately control product selectivity over various reaction conditions. This could be the case for catalysts that incorporate suitable supports or modifiers optimized for various temperature conditions to minimize the degradation of the active sites.

Aside from single Cu atoms and others like Pt and Bi,^27^ NiO is an exciting option to stabilize SnO_2_ because it can be incorporated in much higher proportions using electrospinning.^30,31^ The incorporation of NiO and SnO_2_ goes hand in hand with the possibility of achieving 1D nanofibrous structures to produce non-woven architectures ideal for transport control during electrochemical reactions.^32,33^ Herein, our working hypothesis is that NiO embedding will stabilize the SnO_2_ and increase the hydrogenation activity of SnO_2_, further enabling the formate (HCOO^−^) production. However, it is not ‘*a priori*’ clear how NiO and SnO_2_ combined in an NF can synergistically favor formate production and how temperature affects selectivity. Thus, it is essential to explore.

In this work, we employ electrospinning to synthesize different NiOSnO_2_ NFs by varying Ni : Sn precursor molar ratios between 75 : 25, 50 : 50, and 25 : 75. Among the applied Ni : Sn molar ratios, 50 : 50 and 75 : 25 resulted in an NF-like morphology after annealing at 550 °C while other molar ratios or single compositions did not lead to NFs. Therefore, a detailed structural and morphological characterization (XRD, HR-SEM, and STEM-EDX) is carried out for the 50 : 50 (NiOSnO50NF) and 25 : 75 (NiOSnO75NF) NFs. Additionally, XPS and EELS are used to understand the chemical environment before and after the CO_2_RR. The results show the presence of Ni^3+^ species and partially reduced SnO_2_ after CO_2_ electrolysis at 40 °C. The selectivity of the NFs is evaluated by using an electrochemical flow cell with a maximum heating capacity of 40 °C. The electrochemical experiments for NiOSnO50NF and NiOSnO75NF show HCOO^−^ selectivities close to ∼25% at 25 °C, while at temperatures of 30 °C and 35 °C the HCOO^−^ selectivity increased to ∼30% and ∼50%. An increased HCOO^−^ selectivity is observed at an electroreduction temperature of 40 °C, *ca.* ∼85%, and ∼70% for NiOSnO50NF and NiOSnO75NF, respectively. The product distribution assessed with *in situ* DEMS aligns with the products observed in the flow cell experiments carried out at 40 °C, where, besides methane (CH_4_), HCOO^−^, hydrogen (H_2_), and carbon monoxide (CO) products have also been found. DFT modeling provides insights into the reaction mechanism and the effect of temperature during the CO_2_RR process. Furthermore, the computational surface Pourbaix diagram indicates that combining NiO and SnO_2_ increases the hydrogenation level in the catalyst model, enabling HCOO^−^ production. Our results highlight the importance of catalyst discovery, as demonstrated by the synergistic effects between NiO and SnO_2_, which can boost the electroreduction of CO_2_ at temperatures higher than room temperature.

## 2. Methods

### 2.1 Synthesis of NiO:SnO_2_ nanofibers

The electrospinning technique was used to synthesize metal oxide NFs. For the NiO:SnO_2_ NF, NiCl_2_·6H_2_O (ACS grade, Sigma Aldrich) and SnCl_2_·*X*H_2_O (ACS grade, Sigma Aldrich) were used as precursors. The stock solutions containing Ni, Sn, or their combinations were prepared by dissolving the metal salts in ethanol (100% Tech. grade, BOOM B.V., The Netherlands). Subsequently, polyvinylpyrrolidone (PVP, MW ∼1 300 000 by LS, Sigma Aldrich) is added to the solution and stirred magnetically overnight. The precursor solutions were spun using a commercial electrospinning system from IME Technologies (The Netherlands) at 2.0 mL h^−1^. NFs were obtained at 10.25 kV using a stainless-steel needle of 0.4 mm inner diameter. The collector was maintained at a separation distance of 12 cm from the needle to the aluminum collector plate. The NFs were collected at 22 °C and a relative humidity of 30%. After deposition, NFs were dried in a furnace at 80 °C for 12 h to remove the excess solvent. In a subsequent step, the NFs were annealed in two steps in air. First, the NFs were annealed at 350 °C (ramp-up rate of 1 °C min^−1^) for 3 h to remove the organic components. Second, the NFs were annealed at 550 °C (1 °C min^−1^) for 1 h to produce the NiO:SnO_2_ mixed oxides. Controls were produced using the electrospinning precursor mentioned above. The controls lead to nanoparticles (NP), hereafter labeled as NiOSnONP. In short, the control samples were prepared by directly pouring the electrospinning precursor into porcelain crucibles and calcined at 550 °C (1 °C min^−1^) for 1 h in air.

### 2.2 Morphological, structural and chemical characterization

#### 2.2.1 HR-SEM

High-resolution (HR)-SEM images were taken using a Zeiss MERLIN SEM microscope operated at 1.4 kV coupled with a High-Efficiency Secondary Electron Detector (HE-SE2). Samples were mounted on conductive carbon tape for analysis with no other preparation.

#### 2.2.2 STEM and EELS

Annular Dark Field (ADF) Scanning transmission electron microscopy (STEM) was performed in a JEOL ARM 200 CF system operated at 80 kV. During imaging, the estimated current density was 14.5 pA while using 68–175 mrad of the annular detector's inner and outer angles. The microscope is equipped with an SDD Jeol Centurio Energy-Dispersive X-ray (EDX) spectrometer and a GIF Quantum (Gatan) Dual Electron Energy Loss Spectroscopy (EELS) spectrometer. STEM samples were prepared by dispersing 5 mg of NF sample in ethanol and sonicated for 5 min. The suspension was drop cast on Cu grids.

#### 2.2.3 X-ray diffraction

X-ray powder diffraction was performed in a Bruker D2 PHASER diffractometer, using Cu Kα radiation (*λ* = 1.5418 Å) operated at 30 V, 10 mA, in a range between 2*θ* = 20–85°, employing a step size of 0.05° and a scan speed of 0.1° s^−1^. A low background sample holder (Bruker) was used for the powder samples.

#### 2.2.4 Chemical surface analysis

X-ray photoelectron spectroscopy (XPS) analysis was performed in a Kratos AXIS ULTRA spectrometer using monochromated Al Kα (1486.58 eV). The electron binding energies were referenced to aliphatic carbon C 1s at 284.8 eV. The obtained peak analysis was made using the XPSPEAK41 software. Construction and peak fitting of synthetic peaks in narrow region spectra used a Shirely-type background, and the synthetic peaks were of a mixed Gaussian (30%)–Lorentzian (70%) type.

### 2.3 Electrochemical characterization

#### 2.3.1 Electrochemical flow cell

The electrochemical cell for CO_2_RR consisted of a custom-made microfluidic flow cell using a filter-press configuration. First, catalyst ink was made to prepare the working electrodes. The catalyst ink consisted of a mixture of 3 mg of multiwalled carbon nanotubes (MWCNTs), 7 mg of catalyst material, 228 μL of 5 wt% Nafion perfluorinated solution (Nafion and catalyst + MWCNTs in 1 : 1 ratio by weight) and 600 μL of 2-propanol. Then, MWCNTs are added to the ink to increase the conductivity of the samples. In all samples, the final loading of the catalysts was 1.25 mg cm^−2^, covering a geometrical area of 1.8 cm^2^ of the cathode. The working electrode consists of carbon gas diffusion electrodes (GDE) (SIGRACET 25BC) sprayed with the catalyst ink and dried at 80 °C. Commercial Ir-MMO and leak-free Ag/AgCl electrodes were used as anode and reference electrodes, respectively. The Nafion 117 membrane separates the cathode and anode chambers. The potential was referred to against the reversible hydrogen electrode (RHE).

1 *U*^RHE^ = *U*(Ag/AgCl) + *U*_0_(Ag/AgCl) + 0.059pH

The electroreduction was performed by applying a potential of −0.85 V (*vs.* RHE) for 2 h, using 0.5 M KHCO_3_ as the electrolyte in both chambers, circulated at 23 mL min^−1^. CO_2_ gas was fed at 11 mL min^−1^. The pH value of the CO_2_-saturated electrolyte at 40 °C was 7.9. The different temperatures for electroreduction (25, 30, 35, and 40 °C) were controlled by placing the electrolyte reservoirs in a hot water bath and keeping the cell insulated. The maximum temperature capacity of our electrochemical cell was 40 °C.

An online gas chromatograph (Agilent micro-GC) was connected to the electrochemical cell to analyze the gas products (H_2_ and CO). No CH_4_ was observed, possibly due to the low concentration, and thus, below the detection limit of the micro-GC. After electrolysis, liquid products were analyzed with HPLC (AMinex column HPX-87X from Bio-Rad). The eluent used was 5 mM of H_2_SO_4_ with a 0.6 mL min^−1^ flow rate at 65 °C. Typically, 10 mL of collected catholyte was mixed with 4 M of H_2_SO_4_ to decrease from pH 7.9 to pH 1–3, corresponding to formic acid formation. It should be noted that all current densities are expressed as cathodic currents. Thus, a negative value was used in the manuscript.

The faradaic efficiency (FE) for the products was calculated according to the following equation:2
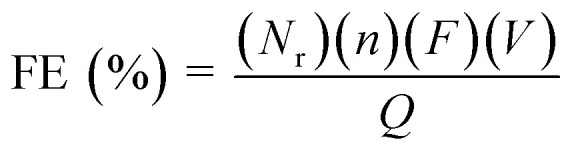
where *N*_r_ is the number of electrons involved in obtaining the product r (2e^−^ for the formate, CO, and H_2_), *n* corresponds to the number of r moles generated, *F* is the Faraday's constant (96 485 C mol^−1^), *V* is the molar flow rate of CO_2_ and *Q* is the total charge passed during electrolysis.

#### 2.3.2 Electrochemical surface area and electrochemical impedance

The electrochemical surface area for the samples was estimated by obtaining the corresponding double-layer capacitance (*C*_dl_) from cyclic voltammetry (CV). CV for the working electrodes in the non-faradaic region was recorded in 0.5 M KHCO_3_, continuously purged with N_2_ at scan rates from 5 mV s^−1^ to 80 mV s^−1^. The *C*_dl_ was estimated for all catalysts from the slope of the linear relationship between the current density in the non-faradaic region and the scan rates in the CV. Electrochemical impedance spectroscopy (EIS) was carried out in the presence of 0.5 M KHCO_3_ as a supporting electrolyte. EIS was done by applying a sinusoidal signal of 10 mV amplitude in the frequency range from 10^5^ to 0.1 Hz at an employed potential of −1.1 V *vs.* SHE in the absence of CO_2_ at 25 °C, while in the presence of CO_2_ at 25 and 40 °C.

#### 2.3.3 *In situ* DEMS

An *in situ* DEMS cell at three electrodes was used to record the ionic current and faradaic current *versus* potential characteristics during cathodic polarization. As for DEMS, the same WE, CE, and RE were employed. The working electrode was prepared on a glassy carbon electrode (3 mm diameter), as described in the previous section. The ionic current (mass signal) for selected mass-to-charge ratios (*m*/*z*) was recorded simultaneously with the CV profiles at a scan rate of 1 mV s^−1^. The CV measurements were conducted in a CO_2_-saturated 0.5 M of KHCO_3_ solution within a potential window from 0.45 to −1.13 V (*vs.* RHE) at 40 °C. The heating increment was made with a heating resistance controlled by an Ink-bird ITC-308-WIFI controller. The ionic current (mass signal) for selected mass-to-charge ratios (*m*/*z*) was also recorded simultaneously with the faradaic current–time profile (*I*–*t*). The electrochemical cell was connected to the quadrupole mass spectrometer (Prisma QMG220) at a working pressure of *ca.* 2.7 × 10^−5^ mbar.

### 2.4 Theoretical methods

#### 2.4.1 First-principles calculations

Spin-polarized Density Functional Theory (DFT) calculations were employed to investigate the structure and thermodynamic stability of SnO_2_(110) models (see 2.4.1.1) using the plane wave Vienna *Ab initio* Simulation Package (VASP).^34,35^ In our samples with and without Ni doping, the SnO_2_(110) surface is typically the most abundantly exposed surface corresponding with 2*θ* = 26.5°.

The generalized gradient approximation (GGA) with Perdew–Burke–Ernzerhof (PBE) exchange–correlation functional was employed to compute the total energies.^36^ The projector-augmented wave (PAW) pseudopotentials were used for the calculations with an energy cutoff of 400 eV for the plane waves.^37,38^ More in particular, 4, 6, 10, and 1 valence electrons were considered for Sn (5s^2^5p^2^), O (2s^2^2p^4^), Ni (3d^8^4s^2^), and H (1s^1^) atoms, respectively. The Grimme-D3(BJ) method was implemented in VASP 5.4.4 to account for the van der Waals interactions.^39,40^ The atomic positions were optimized using the conjugate gradient algorithm with force and electronic convergence criteria of 0.01 eV Å^−1^ and 10^−5^ eV, a Gaussian smearing of 0.05 eV, and a 4 × 3 × 1 Monkhorst–Pack *k*-point grid.^41^ The Partial Hessian Vibrational Analysis (PHVA) was carried out only for the surface species and selected atoms at the active sites on the catalyst (involving three Sn and three bridging O atoms) surface while keeping the other atoms fixed (Fig. S1†). The numerical partial Hessian was calculated by displacing the unfixed atoms in *x*, *y*, and *z*-directions with ±0.01 Å, and the corresponding vibrational modes were obtained by a singular value diagonalization procedure as implemented in the post-processing toolkit TAMKIN.^42^ The zero-point corrections and free energy contributions to the reaction energies were determined from the PHVA-based partition functions to determine the pressure and temperature dependence of the Gibbs free energies.

##### 2.4.1.1 Models

**Fig. 1:**
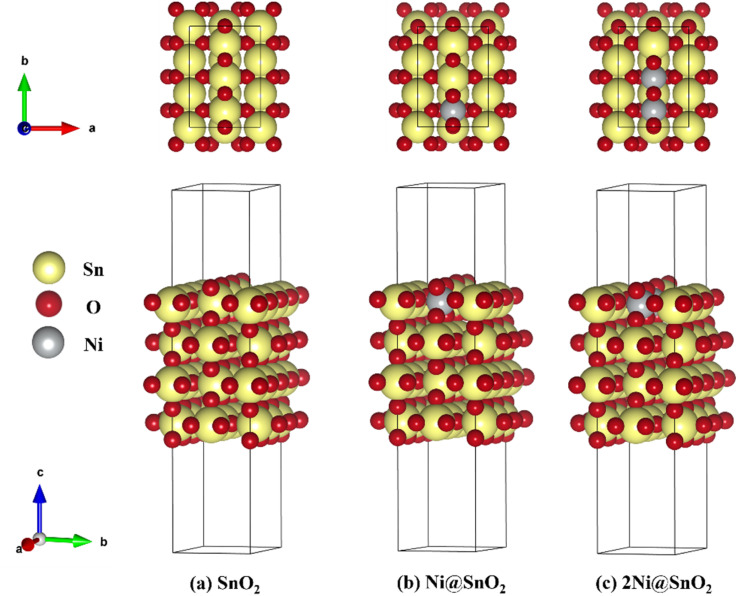
Top and side view of chosen SnO_2_(110) model systems for (a) bare SnO_2_, (b) single Ni-doped SnO_2_ (Ni@SnO_2_), and (c) double Ni-doped SnO_2_ (2Ni@SnO_2_) systems.

The *x*Ni@SnO_2_(110) surface slabs (where *x* = 0, 1, 2) were constructed with 4 SnO_2_ layers, the same number of layers used in previous studies.^43^ We opted for the SnO_2_(110) surface since it was known for its thermodynamic stability and has garnered significant interest in experimental and theoretical investigations.^44–48^ The periodic slab models considered in the study were referred to as SnO_2_, Ni@SnO_2,_ and 2Ni@SnO_2_ (Fig. 1). A vacuum of 15 Å was employed in the *z*-direction to avoid interactions between periodic images. The Ni@SnO_2_ systems were constructed by replacing surface Sn atoms with Ni. To understand the effect of H adsorption on the studied systems during the CO_2_ reduction at the cathode, the 2-fold coordinated O atoms (denoted as Sn–O–Sn) on the surface were hydroxylated, forming Sn–(OH)–Sn. A subsequent H addition to Sn–(OH)–Sn results in adsorbed water bonded to two Sn-sites, *i.e.*, Sn–(OH_2_)–Sn. Additionally, larger supercell models were constructed to shed light onto the interface between NiO and SnO_2_ (see section 15 in ESI for details†).

#### 2.4.2 Surface Pourbaix diagram

Surface Pourbaix diagrams (SPDs) were used to investigate the thermodynamically stable terminations of all studied systems as a function of pH and electrode potential, *U*_SHE_ (Standard Hydrogen Electrode) at pH = 0, *P*_H_2__ = 1 bar, *T* = 26.85 °C, *i.e.*, 300 K.^49^ Model systems up to a maximum coverage of 2H per bridging O were considered for all studied systems to construct the SPDs.

The adsorption of *n* hydrogens on the SnO_2_ surfaces can be given by:

3 *n*(H^+^ + e^−^) + * → *n*H*

where * represents the SnO_2_(110) surface model onto which the hydrogen atoms can be adsorbed.

The change in Gibbs free energy upon *n* hydrogenation reactions with respect to the pristine SnO_2_(110) surface models (Fig. 1) is given by;4
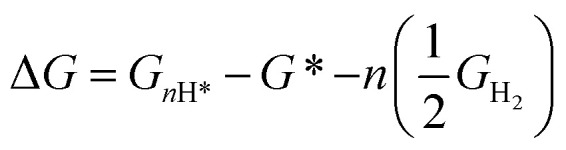


However, to calculate and construct the Pourbaix diagrams, the change in Gibbs free energy corresponding to a sequential hydrogenation reaction is calculated as a function of electrode potential and pH as follows;5
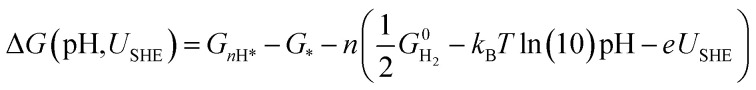
where *G*_*n*H*_ and G_*_ represent the free energy of the pristine and *n* times hydrogen-loaded model systems, respectively. The Gibbs free energy was computed for all terminations, and for a given pH and *U*_SHE_ conditions, the surface termination with the lowest Δ*G*(pH,*U*_SHE_) was considered when constructing the SPD. To compare two competing terminations, the Δ*G*(pH,*U*_SHE_) term is equated, *i.e.*, Δ*G*_A_(pH,*U*_SHE_) = Δ*G*_B_(pH,*U*_SHE_) to determine the pH *vs. U*_SHE_ conditions of the equilibrium lines in the SPD. *G*_nH*_ can be further described in terms of its vibrational contributions:

6 *G*_*n*H*_ = *E*_DFT_ + *E*_ZPVE_ + Δ*E*_vib_(*T*) − *TS*_vib_ ≈ *E*_DFT_ + *F*_vib_(*T*)

Here, *E*_DFT_ represents the total energy while *F*_vib_ is the Helmholtz vibrational energy. For the gas phase molecules, the thermodynamic quantities such as ZPE, 
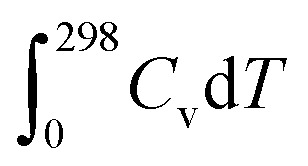
, *TS*, were obtained from the ideal gas approximations from a previous report (Table S4†). The Gibbs free energy for the gas molecules at 25 °C (*i.e.*, 298 K) and 1 atm is given as:7
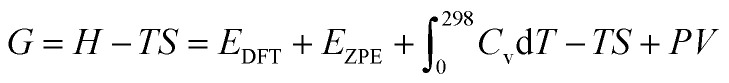
where *E*_DFT_, *E*_ZPE_, 
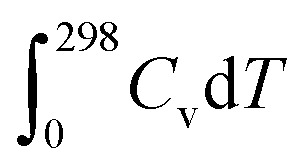
 and *S* denote the electronic energy, zero-point vibrational energy, heat capacity, and entropy, respectively.

## 3. Results and discussions

### 3.1 Synthesis of NiO:SnO_2_ nanofibers

Electrospinning is used to produce NiO:SnO_2_ NFs. NFs are synthesized in various compositions, changing the molar ratios between Ni and Sn, *i.e.*, 75% : 25% (NiOSnO25NF), 50% : 50% (NiOSnO50NF), and 25% : 75% (NiOSnO75NF). Similarly, NiO and SnO_2_ single compositions have been electrospun. However, NiO and SnO_2_ do not lead to NFs under similar electrospinning conditions. The latter includes NiO and SnO_2_ formulations, which lose the NF shape after annealing. For instance, a single electrospun composition of NiO and SnO_2_ NF can lead to the formation of nanoparticles after annealing (Fig. S2†). Like NiO and SnO_2_, NiOSnO25NF led to nanoparticle formation after annealing (Fig. S3†). The previous results indicate that the spun NFs could not retain the fiber shape during annealing due to the unstable formation of metal oxide, leading to NF-shape coalescence. Similar results have been observed for NiO systems, indicating that our NiCl_2_-PVP formulation leads to nanoparticle (NP) upon annealing.^12^ This can also be the case for SnO_2_, which could occupy additional metal oxide agents to maintain the NF shape.^50^ Due to the lack of NF shape, NiOSnO25NF has not been analyzed further. However, NiO and SnO_2_ are still used as controls, along with NiOSnO50NP and NiOSnO75NP, which both lack NF shape.

**Fig. 2:**
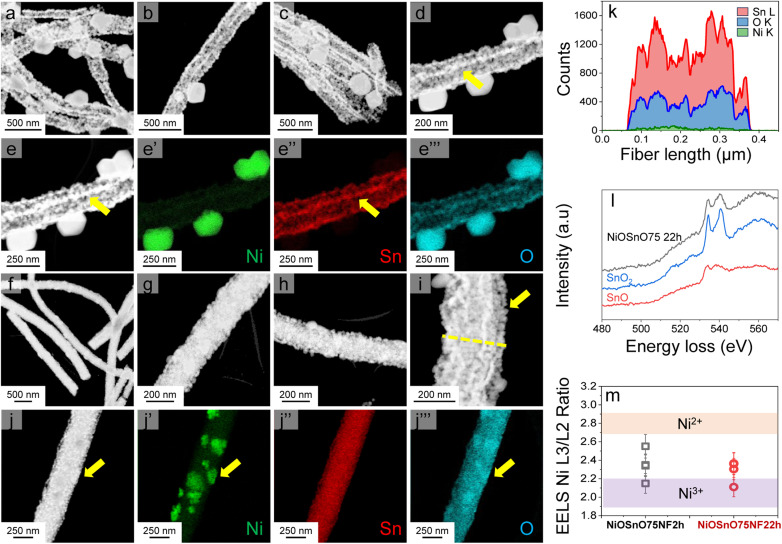
STEM-ADF images (a–e) and STEM-EDX maps (e′ and e′′′) for NiOSnO50NF. STEM-ADF images (f–j) and STEM-EDX maps (j′ and j′′′) for NiOSnO75NF. The STEM-EDX line scan for NiOSnO75NF22h is shown in (k). EELS measurements NiOSnO75NF22h are shown in (l) and (m) for Sn and Ni, respectively. SnO_2_ (blue line) and SnO (red line) controls are presented in (l). In (m), the L3/L2 ratio of Ni L_23_ edge EELS for NiOSnO75NF2h and NiOSnO75NF22h are carried out over different surface areas of the crystallites.

NiOSnO50NF and NiOSnO75NF are inspected with STEM-ADF and STEM-EDX, as shown in Fig. 2. For NiOSnO50NF, Fig. 2a–d displays an NF-like shape with an NF diameter of 209 ± 45 nm. The NiOSnO50NF comprises ∼50 nm nanocrystals and multiple ∼10 nm nanocrystals or smaller in diameter. The inner structure corresponds to a polycrystalline arrangement of nanoparticles with some gaps between them. Interestingly, in some cases, a row of nanocrystals forms a string along the NF with higher contrast (*e.g.*, Fig. 2d and e, yellow arrows). From STEM-EDX mapping Ni, Sn, and O (Fig. 2e′ and e′′′), we have identified that these nanoparticle rows primarily comprise oxidized Sn. The contrast over the rows is attributed to densified oxidized Sn. In addition, within the NiOSnO50NF, oxidized Ni has been found to a lesser extent over the NF body; however, larger NiO particles, typically ∼200 nm in diameter, decorate the NF morphology. A closer look at the interface between the NiO:SnO_2_ nanocrystallites is discussed in Fig. S4.† For NiOSnO75NF in Fig. 2f–i, the NF is more compact than in NiOSnO50NF, where nanocrystals are relatively smaller with a 35 nm diameter or less (*e.g.*, Fig. 2i, yellow arrow). The presence of nanocrystals along the NF shape forming bright strings is less evident than in NiOSnO50NF. Moreover, NiO forms patches over the NF morphology (*e.g.*, Fig. 2j, yellow arrow). The STEM-EDX mapping for Ni, Sn, and O in Fig. 2j′ and j′′′ supports these results (see yellow arrows). The presence of Ni within the NF morphology is also verified by the STEM-EDX line scan (Fig. 2i and k, see dashed yellow line). The STEM-EDX line scan demonstrates that the Ni remains within the NF body but to a lower extent when compared to Sn (Fig. 2k). The NiOSnO75NF chemical species have also been investigated with EELS after CO_2_ electroreduction (Fig. 2l and m). In Fig. 2l, the EELS signals for NiOSnO75NF after 22 h of CO_2_ electroreduction (NiOSnO75NF22h, black line) show that the oxidized tin resembles SnO_2_ (blue line). However, we should not disregard the possibility of partially reduced SnO_2_ since the NiOSnO75NF22h has some similarities to SnO (red line). It is then suggested that NiOSnO75NF22h contains multiple oxidized Sn species (SnO_*x*_). In Fig. 2m, the Ni L23 edge is used to obtain the L3/L2 ratio and determine the chemical environment of Ni-species in NiOSnO75NF after 2 h of CO_2_ electroreduction (NiOSnO75NF2h) and NiOSnO75NF22h. The EELS L3/L2 ratio shows that for NiOSnO75NF2h, more points have a wider L3/L2 ratio,^12^ indicating a higher fraction of Ni^2+^ and Ni^3+^ species than NiOSnO75NF22h, which reveals a narrower L3/L2 ratio, close to Ni^3+^ species. The results denote that uncoordinated Ni^3+^ species, *e.g.*, defects, are formed more due to longer electrolysis time attributed to the loss of NF shape after CO_2_ electrolysis.^12^

**Fig. 3:**
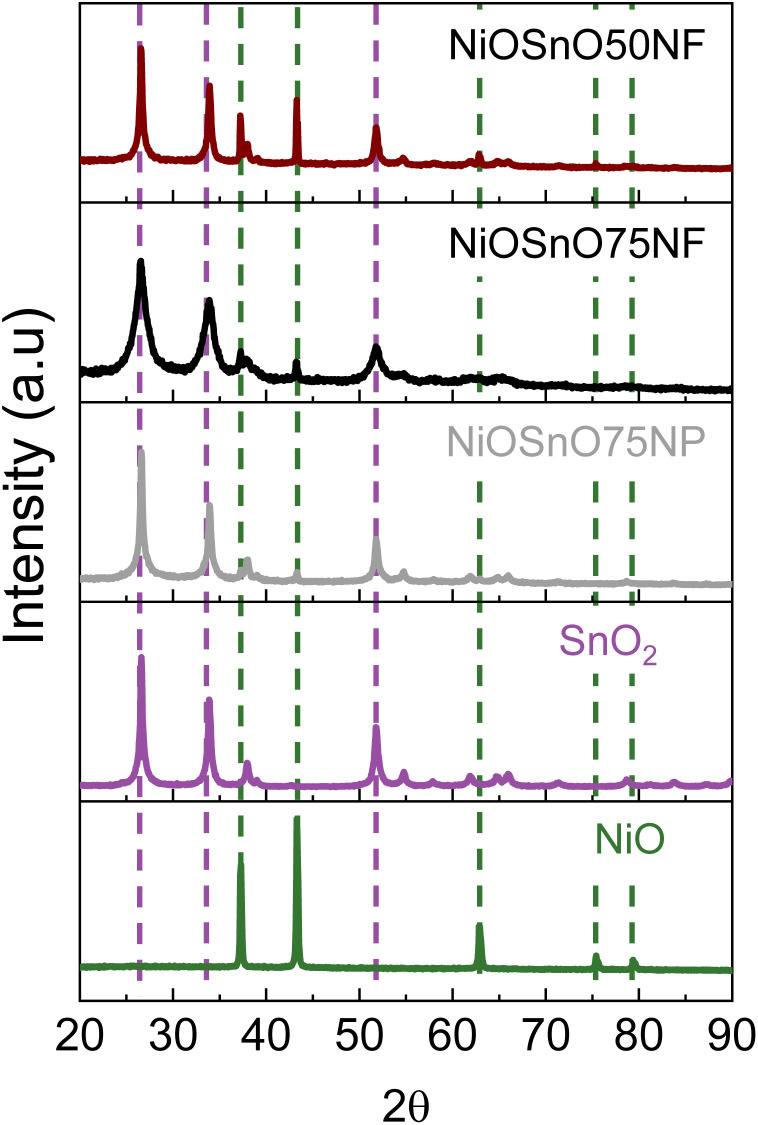
XRD diffraction patterns for NiOSnO50NF and NiOSnO75NF. XRD diffraction patterns of NiOSnO75NP, SnO_2_, and NiO are included for controls.

From the STEM-EDX results in Fig. 2, we can verify the presence of metal oxides. To confirm the oxide type, we look at the structural characteristics of NiOSnO50NF and NiOSnO75NF using XRD (Fig. 3), with NiO and SnO_2_ as controls. First, we describe the diffractograms of NiO and SnO_2_. NiO has several diffraction peaks at 2*θ* = 37.2°, 43.3°, 62.9°, 75.4°, and 79.3°, which correspond to (111), (200), (220), (311), (222) crystallographic planes from NiO (JCPDS 65-6920), respectively.^51^ SnO_2_ also shows diffraction peaks at 2*θ* = 26.5°, 33.9°, 37.9°, 51.8°, 54.6°, 57.8°, 61.8°, 64.7°, 66.0°, 71.3°, and 78.7°, corresponding to (110), (101), (200), (211), (220), (002), (310), (112), (301), (220), and (321) crystallographic planes from SnO_2_ respectively (JCPDS No. 41-1445).^52–54^ Comparing NiO and SnO_2_ with NiOSnO50NF and NiOSnO75NF, we observed that the crystallographic phases correspond to NiO and SnO_2_. No changes in the diffraction peak positions for NiOSnO75NP used as control are observed. No evidence of a difference in crystallographic phase has been found, indicating that NiO and SnO_2_ prevail in separate phases within the NF (Fig. 2 and 3).

**Fig. 4:**
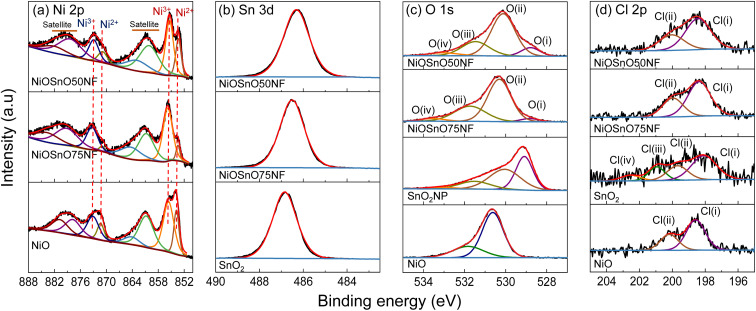
(a) Ni 2p, (b) Sn 3d, (c) O 1s, and (d) Cl 2p XPS core spectra for NiOSnO50NF, NiOSnO75NF, NiO, and SnO_2_.

The chemical environment is investigated with XPS to determine the type of species present over NiOSnO50NF and NiOSnO75NF. The XPS core spectra of Ni 2p, Sn 3d, O 1s, and Cl 2p for NiOSnO50NF and NiOSnO75NF are presented in Fig. 4. NiO and SnO_2_ controls are used for comparison. In Fig. 4a, Ni 2p comprises Ni 2p_3/2_ and Ni 2p_1/2_. The Ni 2p_3/2_ peak can be fitted into two components corresponding to Ni^2+^ and Ni^3+^ species, labeled in red. Ni^2+^ and Ni^3+^ peaks are located at 853.8 eV and 855.6 eV.^55–57^ The presence of Ni^3+^ can be ascribed to the uncoordinated species, like defects.^12^ The Ni^3+^/Ni^2+^ ratio for NiOSnO50NF is estimated to be close to 2.1, while NiOSnO75NF is around 8.5, indicating that Ni^3+^ is more prominent in NiOSnO75NF. Similar Ni^3+^/Ni^2+^ has been observed for NiOSnO50NP and NiOSnO75NP compared to the NF counterpart. Furthermore, a behavior opposite to NiOSnO50NF and NiOSnO75NF has been observed for NiO, in which the Ni^3+^/Ni^2+^ ratio is lower, *ca.* 1.3. Ni^2+^ and Ni^3+^ species have also been identified with EELS (Fig. 2), supporting our finding. The results demonstrate that the presence of Sn increases the amount of Ni^3+^ species. Next, we analyze the results for Sn 3d. In Fig. 4b, NiOSnO50NF and NiOSnO75NF show binding energy (BE) for Sn 3d_5/2_ around 486.3–486.5 eV, assigned to Sn^4+^ in SnO_2_.^58–60^ Compared to SnO_2_ control with a BE around 486.9 eV associated with Sn^4+^,^61^ a shift to lower BE has been found for NiOSnO50NF and NiOSnO75NF. This shift can be related to reduced Sn species (*e.g.*, SnO_*x*_), similar to EELS, as shown in Fig. 2l.

The XPS core spectra of O 1s and Cl 2p and the fitting curves have also been analyzed (Fig. 4c and d). O 1s core spectra for NiOSnO50NF and NiOSnO75NF show four different peaks resulting from NiO and SnO_2_ formation within the NF body. The O(I) peak (BE 528.8–529.1 eV) is attributed to oxygen in NiO.^55,56^ The O(II) peak (BE 530.0–530.3 eV) is attributed to mixed oxygen species from NiO and SnO_2_.^57,62,63^ The O(III) peak (BE 531.3–531.7 eV) is attributed to surface OH groups.^56,63,64^ The O(IV) peak (BE 532.9–533.5 eV) is attributed to chemisorbed water.^63,64^ Similar results have been obtained for NiO. The O 1s XPS spectrum of NiO shows BE at 529.1 eV, 530.0 eV, 531.5 eV, and 533.3 eV, attributed to oxygen in NiO,^55,56^ surface O^2−^ species,^65,66^ surface OH groups, possibly from uncoordinated Ni^3+^ species present in NiO.^12^ As for SnO_2_, the O 1s XPS spectrum shows BE at 530.6 eV and 531.8 eV, corresponding to oxygen in SnO_2_ (ref. 67–69) and OH groups.^68,69^ Cl 2p core spectra for NiOSnO50NF, NiOSnO75NF, NiO, and SnO_2_ show several peaks labeled as Cl(I), Cl(II), Cl(III), and Cl(IV). Cl(I) between 198.6–198.1 eV and Cl(II) between 199.7–200.2 eV are assigned to inorganic chlorine species.^70–73^ NiO reveals two additional peaks at Cl(III) at 200.9 eV and Cl(IV) at 202.5 eV, both corresponding to Cl^−^ from different decomposed chemical species of chlorine salt.^12,74^ The concentration of Cl^−^ for all the samples remains similar, with an average atomic percentage of 1% ± 0.2.

### 3.2 CO_2_ electroreduction

**Fig. 5:**
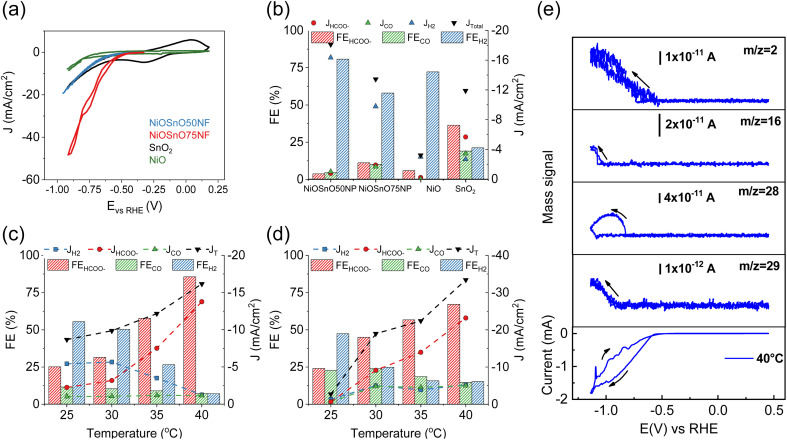
(a) CV characteristics of NiOSnO50NF, NiOSnO75NF, NiO, and SnO_2_ at 25 °C. FEs and partial current densities for (b) NiOSnO50NP, NiOSnO75NP, NiO, and SnO_2_ at 40 °C, along with (c) NiOSnO50NF and (d) NiOSnO75NF at −0.85 V *vs.* RHE for 2 h over various temperatures, *i.e.*, 25, 30, 35, 40 °C. (e) CV characteristic for NiOSnO75NF at 40 °C. *In situ* DEMS mass signals recorded at 40 °C are shown as a function of the applied potential (1 mV s^−1^) for *m*/*z* = 2, *m*/*z* = 16, *m*/*z* = 28, and *m*/*z* = 29. In all cases, the pH of the bulk electrolyte remained at 7.9.

The functionality of NiOSnO50NF and NiOSnO75NF for CO_2_RR is assessed in Fig. 5. To elucidate the effect of NF functionality, NiOSnO50NF and NiOSnO75NF are compared to NiOSnO50NP and NiOSnO75NP, which lack the NF shape. Additionally, the results are contrasted with NiO and SnO_2_. The NiOSnO50NF, NiOSnO75NF, NiOSnO50NP, NiOSnO75NP, NiO, and SnO_2_ comparatives are conducted in a flow cell to demonstrate the importance of the synthesized multimetal oxide NFs. Finally, *in situ* DEMS experiments are discussed to shed light on the product pathways.

The experiments start with preparing the CO_2_RR GDEs by spraying with ink containing MWCNTs, catalyst, and Nafion. The dried GDE is placed in the flow electrochemical cell using a three-electrode configuration containing a solution of 0.5 M KHCO_3_ as an electrolyte. The CV in the presence of CO_2_ shows the highest current density (*J*, mA cm^−2^) for the NiOSnO75NF, followed by NiOSnO50NF and SnO_2_, with NiO showing the lowest in *J* (Fig. 5a). At 40 °C and −0.85 V *vs.* RHE, the product distribution for NiOSnO50NP, NiOSnO75NP, NiO, and SnO_2_ are also evaluated (Fig. 5b). It should be noted that three different potentials have been used, *i.e.*, −0.75, −0.85, and −0.95 V *vs.* RHE, and −0.85 V is selected since it produced the highest FE_HCOO^−^_ and *J*_HCOO^−^_. Compared to Fig. 5c and d, the results highlight the advantage of the synthesized NFs.

In Fig. 5b, FE for NiOSnO50NP and NiOSnO75NP at 40 °C displays the product distribution, where HCOO^−^, H_2_, and CO are formed during CO_2_ electroreduction. The FE for HCOO^−^, H_2_, and CO for NiOSnO50NP are FE_HCOO^−^_ = 3.8%, FE_H_2__ = 80.7% and FE_CO_ = 4.7% with partial *J* values of *J*_HCOO^−^_ = −0.7 mA cm^−2^, *J*_H_2__ = −14.7 mA cm^−2^ and *J*_CO_ of = −0.9 mA cm^−2^. Similar product distribution is observed for NiOSnO75NP with FE_HCOO^−^_ = 11.2%, FE_H_2__ = 58.0%, and FE_CO_ = 10.1% have been found with *J*_HCOO^−^_ = −1.7 mA cm^−2^, *J*_H_2__ = −8.8 mA cm^−2^ and *J*_CO_ = −1.5 mA cm^−2^. For NiO, a prominent generation of H_2_ is observed with FE_H_2__ = 72.2% and *J*_H_2__ = −2.7 mA cm^−2^. For the same electrocatalyst, FE_HCOO^−^_ = 6% and *J*_HCOO^−^_ = −0.2 mA cm^−2^, no CO has been detected. It could be argued that to increase HCOO^−^ formation, NiO should be selectively shaped as octahedra and not as mixed particle shapes (Fig. 2).^12^ SnO_2_ FE for HCOO^−^, H_2_, and CO are 37%, 21%, and 19% with *J*_HCOO^−^_ = −5.1 mA cm^−2^, *J*_H_2__ = −2.4 mA cm^−2^ and *J*_CO_ of = −3.1 mA cm^−2^. The results demonstrate the importance of synergistic effects between NiO and SnO_2_, particularly for the NF morphology (Fig. 5c and d).

The effect of the temperature (25, 30, 35, and 40 °C) during CO_2_R for NiOSnO50NF and NiOSnO75NF is evaluated in Fig. 5c and d. For NiOSnO50NF (Fig. 5c), the HCOO^−^ has a gradual increase in selectivity as the temperature increases, starting from FE_HCOO^−^_ = 25.2% at 25 °C and reaching a maximum of FE_HCOO^−^_ = 85.7% at 40 °C. At 40 °C, the highest *J*_HCOO^−^_ is observed (−13.8 mA cm^−2^). For FE_H_2__, we observe a gradual decrease in H_2_ generation as a function of temperature, starting from FE_H_2__ = 55.4% at 25 °C and reaching a minimum of 7% at 40 °C. The *J*_H_2__ value for NiOSnO50NF follows a similar trend, from *J*_H_2__ = −5 mA cm^−2^ at 25 °C, reaching a minimum *J*_H_2__ = −1.2 mA cm^−2^ at 40 °C. FE_CO_ does not drastically decrease with increasing temperature, maintaining *J*_CO_ around −1 mA cm^−2^ across the various conditions, with a FE_CO_ = 7.2% at 40 °C. Overall, in NiOSnO50NF, FE_HCOO^−^_ is favored as temperature increases while maintaining FE_CO_ constant and suppressing FE_H_2__. NiOSnO50NF and NiOSnO75NF increase the total current densities (*J*_T_) with temperature.

Next, the electrochemical performance of NiOSnO75NF during CO_2_ electroreduction is discussed (Fig. 5d). For HCOO^−^, a gradual increase in selectivity, with FE_HCOO^−^_ = 25% at 25 °C and FE_HCOO^−^_ = 70% at 40 °C, is observed. At 40 °C, the highest *J*_HCOO^−^_ is observed with −26.1 mA cm^−2^. The FE_H_2__ in NiOSnO75NF also follows a gradual decrease with increasing temperature, with FE_H_2__ = 47.4% at 25 °C and 15.2% at 40 °C. For H_2_, *J* remains at *J*_H_2__ = −5 mA cm^−2^ from 30 °C to 40 °C. Lastly, FE_CO_ presents a gradual decrease with 22.6% at 25 °C and 14.5% at 40 °C with *J*_CO_ = −5 mA cm^−2^ for temperatures similar to or higher than 30 °C. Although FE_HCOO^−^_ for NiOSnO75NF remained 15% lower than for NiOSnO50NF at 40 °C, NiOSnO75NF has a 2-fold increase in *J*_HCOO^−^_. This 2-fold increase can be attributed to an increase in the electrochemical surface area (ECSA) as the obtained double-capacitance is higher for NiOSnO75NF (4.68 × 10^−4^ mF cm^−2^) than NiOSnO50NF (3.78 × 10^−4^ mF cm^−2^). Furthermore, the results at 40 °C for NiOSnO75NF are substantiated with EIS. EIS reveals less charge transfer resistance and an increased affinity in the presence of CO_2_ for NiOSnO75NF (Fig. S5 and Table S5†). Likewise, there is no significant effect when looking at the Tafel slopes in the presence of CO_2_ (Fig. S6 and Table S6†). The Tafel slopes are somehow similar. Hence, the results indicate the existence of similar rate-determining steps in the presence of CO_2_ for temperatures close to 40 °C. Slight variations in the Tafel slopes are observed for temperatures close to 45 °C, suggesting a different rate-determining step associated with other processes, *e.g.*, H_2_ competition. The results of the chronoamperometry from Fig. 5c and d are shown in Fig. S7.†

In short, a trade-off between selectivity and product yield should be found when assessing catalyst performance. However, NiOSnO50NF and NiOSnO75NF resulted in similar trends, elucidating temperature effects, which could be reasonably associated with favored reaction kinetics at high temperatures.^19–21^ Such effects have not been observed during CO_2_ electroreduction using synergistic catalysts shaped as NFs. Hence, the synergistic effects require an understanding of the reaction product to the fullest. Therefore, an *in situ* DEMS is assessed to generate insight into the reaction product pathway by detecting the formic acid (HCOOH) mass fragments for NiOSnO75NF, as it yielded the highest HCOO^−^ production at 40 °C (Fig. 5d). Mass spectrometric signals corresponding to H_2_ (*m*/*z* = 2), methane (CH_4_, *m*/*z* = 16), CO (*m*/*z* = 28), and HCO^−^ (*m*/*z* = 29) from HCOOH,^75^ and CV are recorded simultaneously (Fig. 5e). It should be noted that mass *m*/*z* = 29 is selected as it is the most pronounced for HCOOH, and in the absence of CO_2_, no CO_2_ reaction products are observed (Fig. S8†). Additionally, to corroborate the detection of HCOOH, formic acid is added to the electrolyte, and the mass signals associated with this organic compound are shown in Fig. S9.† Overall, the distribution of DEMS products confirmed our flow cell observations in Fig. 5d, except for CH_4_, which could be expected to be below the detection limit of our gas chromatograph but captured by DEMS. It should be noted that other factors that might change reaction product selectivity to CH_4_ can be related to the DEMS cell configuration as it can impact pH, generating some gradients.^75^

Lastly, we discuss the effect of uncoordinated Ni species found in NiOSnO NFs, which could have enabled the formation of HCOO^−^.^12^ We could expect NiO species to enhance hydrogenation over SnO_2_, which is more likely to be as partially reduced SnO_2_, *i.e.*, SnO_*x*_, after 2 or 22 h CO_2_ electrolysis. Although at 2 h, the NF shape drastically changed its morphology (Fig. S10†), Ni and Sn species remained present even after 22 h of CO_2_ electrolysis (Fig. 2l and m). Furthermore, the Ni^3+^/Ni^2+^ ratios for NFs connect with the improved FE_HCOO^−^_ at 40 °C. However, we should not disregard the Ni^3+^/Ni^2+^ ratio in NPs. For example, the Ni^3+^/Ni^2+^ ratio for NiOSnO75NP is 7.5, close to NiOSnO75NF (*i.e.*, 8.5). The benefit of structuring becomes evident when comparing the Raman spectra in Fig. S11† for NiOSnO75NP and NiOSnO75NF. NiOSnO75NP contains more organic species than NiOSnO75NF, compromising the CO_2_ reduction reaction activity (Fig. 2b and d).

The results highlight the advantage of the NF morphology as carbon is removed from the NiOSnO precursor due to the open fibrous structure. Similar effects have been observed for polymer-derived metal oxides, such as 3D-printed structures where carbon remnants are found.^76^ Hence, the carbon remnant could act as a blocking layer during CO_2_RR, affecting NiOSnO75NP selectivity. This hypothesis is well aligned with NiOSnO75NF loaded with *t*-octylphenoxypolyethoxyethanol (Triton ×100) used as a surfactant, acting as a carbon-blocking agent without compromising the NF morphology after annealing (Fig. S12†). The electrochemical results of NiOSnO75NF loaded with surfactant demonstrate a change in the product distribution with low HCOO^−^ selectivity over the explored temperature ranges (Fig. S13†). The results are substantiated further by ECSA. ECSA result for NiOSnO75NF is 4.68 × 10^−4^ mF cm^−2^, while in the presence of a surfactant or NiOSnO75NP, it decreases to 8.39 × 10^−5^ mF cm^−2^. Now that we have identified the importance of blocking agent-free catalysts, we propose a mechanism for the NiOSnONF using the most significant products (H_2_ and HCOO^−^), as shown in Fig. 5c and d.

### 3.3 CO_2_RR mechanism

#### 3.3.1 Surface Pourbaix diagrams

**Fig. 6:**
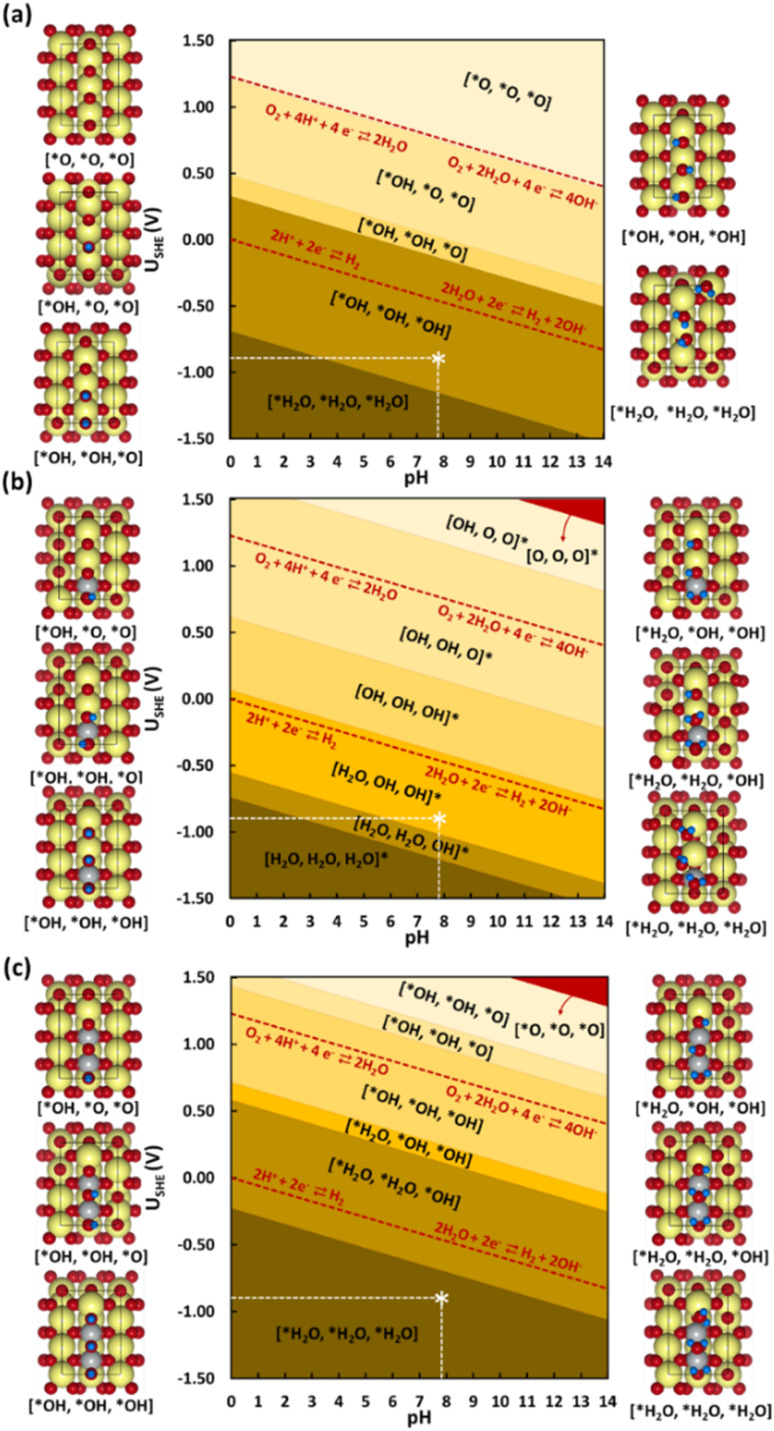
Surface Pourbaix diagrams for the studied (110) surface models (a) SnO_2_, (b) Ni@SnO_2_, and (c) 2Ni@SnO_2_. Surfaces with no H adsorbed are colored in red. Color codes: Sn (yellow), O (red), H (blue), and Ni (grey). The white star * in the diagram corresponds to typical experimental conditions used (*U*_SHE_ = 0.85 V, pH = 7.9).

Before deriving the CO_2_RR mechanism, evaluating the stability of the electrocatalyst and its surface termination under electrochemical conditions is essential. The ESI in Section 14, Table S7,† describes the stability of the electrocatalyst models in terms of their cohesive and surface formation energy. For models with an increasing Ni concentration in the SnO_2_ surface, the cohesive and surface formation energies become more negative, indicating a favorable formation of Ni-doped SnO_2_ phases. For the pristine NiO, the cohesive energy is more positive (0.161 eV per atom) compared to the Ni-doped SnO_2_ systems (0.061 eV per atom and −0.040 eV per atom, respectively). SPDs have been shown to play a crucial role in elucidating thermodynamically stable terminations as a function of pH and electrode potential.^12,77–79^ The calculated SPDs at the experimental electrochemical conditions are shown in Fig. 6. The NiO:SnO_2_ interface and stability *vs.* potential diagram for all models at pH = 0 is displayed in Fig. S14–S17.† In an aqueous environment, the surfaces and active sites tend to hydroxylate (Fig. 6). Fig. 6a–c show the SPDs for the studied SnO_2_(110) model systems with and without Ni doping, which all have 3 bridging oxygens represented by [*O, *O, *O]. In the following, hydrogen coverage refers to these three bridging oxygens, and therefore, 1 ML hydrogen coverage corresponds with the system represented as [*OH, *OH, *OH]. In the potential range of −1.5 to 1.5 V, five different terminations are found in the SPD of the studied pristine SnO_2_(110) surface model (Fig. 6a). Above 1.21 V and low pH, the configuration with no adsorbed H (or H*) is stable. As the potential decreases, the adsorption of H gets pronounced, leading to a complete H* coverage of the bridging O atoms. For instance, between 1.21 V and 0.487 V at pH = 0, the termination with 0.33 ML H* is favored. The other stable terminations are 0.66 and 1.00 ML of H*. Following the hydration of the oxygens bridging two Sn atoms (Sn–O–Sn), H can further adsorb on the bridging hydroxyls, forming water molecules. The termination with 2.00 ML of H [*H_2_O, *H_2_O, *H_2_O] is favored at potentials lower than −0.69 V. This also implies that the SnO_2_ tends to reduce at cathodic electrode potentials, thus forming stable reduced surfaces which can be active for catalysis. The dashed line in red represents the standard OER and HER limits.

Next, the influence of Ni-doping of the SnO_2_(110) model system on the Pourbaix diagram is depicted in Fig. 6b and c. Unlike for SnO_2_, all H-covered terminations from 0.33 to 2.00 ML H* are present in the Pourbaix diagram for Ni-doped SnO_2_ models at potentials between −1.5 and 1.5 V. In the case of a single Ni-doped SnO_2_(110) system, the surface with no H adsorbed is stable only at higher potentials (>2.14 V) and pH (>13). Between 1.5 and 0.06 V (at pH = 0), the two-fold bridging O* atoms (Sn–O–Sn) tend to get fully hydrogenated. At lower potentials (<0.06 V), the hydrogenated O atoms can adsorb H to form adsorbed water molecules. Below −0.74 V *versus* RHE, the surface is completely reduced with all two-fold bridging O atoms (Sn–O–Sn) forming water molecules. As a characteristic of H adsorption, on moving to a higher pH, the stable H terminations occur at lower potentials in the SPD due to the shift of −59 meV per pH unit. Finally, another Sn atom is replaced with Ni to understand the effect of Ni concentration on the SPDs. For the 2Ni@SnO_2_ surface model, all H terminations from 0.33 to 2.00 ML H* appeared in the Pourbaix diagram. The surface with no hydrogen appears only above 2.10 V and high pH. The termination with 1.00 ML coverage of H is favorable under OER conditions, while the surface with 1.66 ML H* coverage (2× H_2_O*) appears at the HER limit. Interestingly, at the experimental conditions of *U* = −0.85 V and pH = 7.9 (Fig. 5), the surface with 2.00 ML (3× H_2_O*) is likely to be thermodynamically preferred. This also highlights that in CO_2_RR conditions, the surface of the Ni-doped SnO_2_ is partially reduced (*e.g.*, SnO_*x*_, Fig. 2 and 4), which could further tailor the electrocatalytic activity at the surface.^80,81^ Overall, the SPDs emphasize the reduction of the SnO_2_-based catalyst surface, specifically the co-adsorption of water molecules at potentials of experimental interest.

#### 3.3.2 CO_2_RR mechanism

The SPDs allow us to determine the relevant active site model termination under operating conditions, and subsequently, CO_2_ reduction pathways are determined. A typical reduction of CO_2_ to HCOO^−^ (*i.e.*, hereafter HCOOH) over SnO_2_ occurs *via* a 2e^−^ pathway with the elementary steps described as:

8 CO_2_ + * → *CO_2_

9 *CO_2_ + H^+^ + e^−^ → *HCOO

**Fig. 7:**
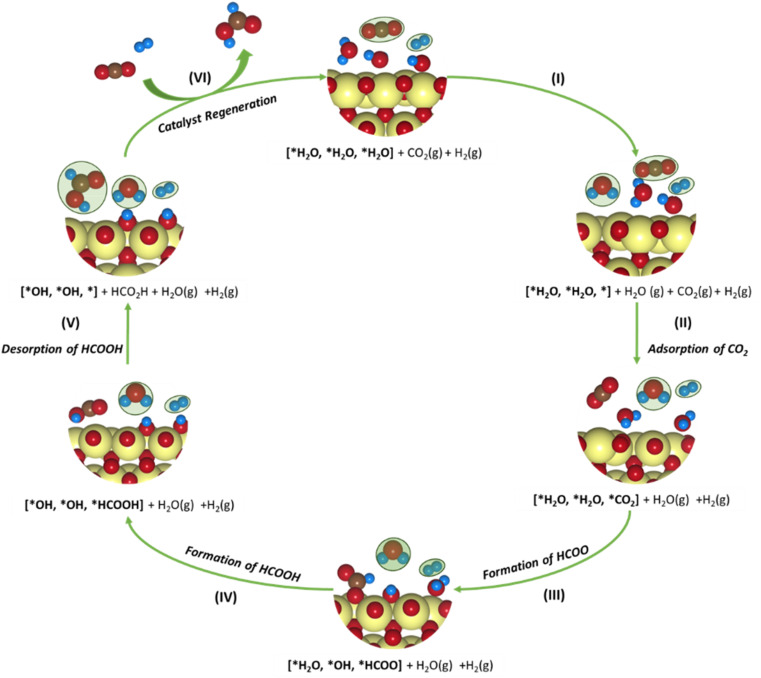
Schematic representation of the plausible CO_2_ reduction mechanism for the SnO_2_-based model systems. The adsorbates in gaseous states are marked in green. Color codes: Sn (yellow), C (brown), O (red), H (blue).

**Fig. 8:**
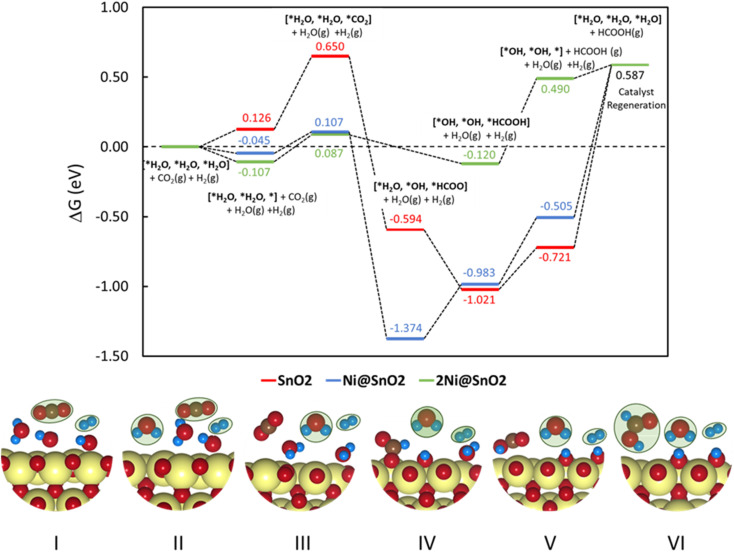
Free energy diagram for CO_2_ reduction to HCOOH over SnO_2_(110), Ni@SnO_2_(110), and 2Ni@SnO_2_(110) electrocatalyst models. (Bottom panel) The binding modes of the adsorbates on the catalyst at different reaction states and the adsorbates in the gaseous state are marked in green. Color codes: Sn (yellow), C (brown), O (red), H (blue).

10 *HCOO + H^+^ + e^−^ → HCOOH + *

where * denotes the active site of the catalyst. Although the proton-coupled electron transfer (PCET) mechanism assuming steps with H^+^/e^−^ pairs (equivalent to ½H_2_) is vastly employed in the context of DFT-based studies,^43,48^ it usually oversimplifies the catalytic surface in which protons can be supplied from the dissociation of water molecules at the electrode–electrolyte interface. T. Burdyny and W. A. Smith demonstrated that at current densities above 35 mA cm^−2^, the proton for CO_2_ reduction is supplied by the water molecules on the electrode surface, increasing the local interfacial pH.^82^ The resulting change in the local environment further influences the binding energies of intermediates and surface coverage on the electrode. The SPDs discussed previously highlighted this, showing that the bridged oxygen forms water molecules at lower potentials, resulting in an overall reduction of the catalytic surface. Fig. 7 presents a plausible mechanism for the CO_2_RR to HCOO^−^ for the SnO_2_-based models involving a PCET reaction considered in the study. It can be found that the active catalyst surface of 2Ni@SnO_2_ is covered with 2.00 ML of H (*i.e.*, three H_2_O per unit cell), which is represented as [*H_2_O, *H_2_O, *H_2_O] following its SPD. This model could approximate the local environment on the reduced surface more appropriately than the usual models that do not consider electrolyte species' co-adsorption. Although the three water molecules do not appear in the SPDs of SnO_2_ and Ni@SnO_2_ at the experimental conditions of −0.85 V and a pH of 7.9, we consider also 2.00 ML coverage models for comparison with the 2Ni@SnO_2_ system (Fig. 8).

**Fig. 9:**
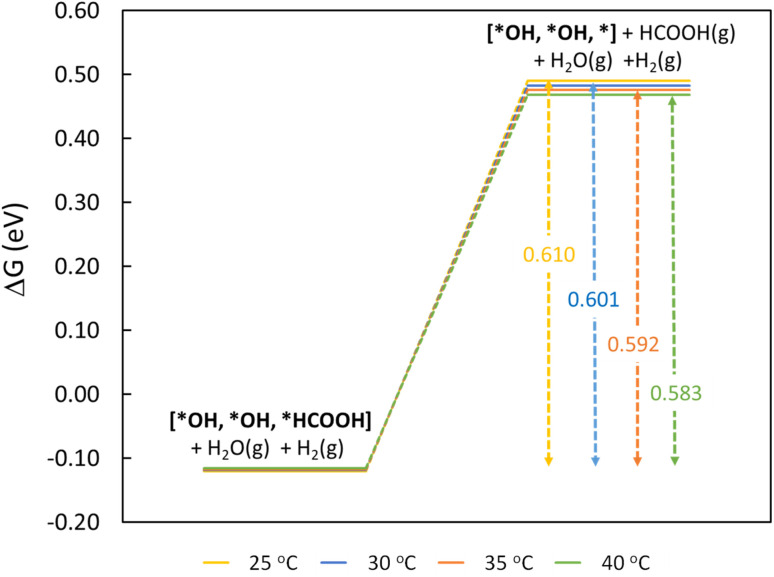
Free energy diagram for rate-limiting step of HCOOH desorption as a function of temperature on the 2Ni@SnO_2_(110) model.

To understand the thermodynamic feasibility of the reaction pathway proposed, we calculated the Gibbs free energy profile for each model system. The mechanism starts with the desorption of one of the H_2_O molecules on 2Ni@SnO_2_, leaving an empty site for CO_2_ adsorption [*H_2_O, *H_2_O, *] (Fig. 8). From Fig. 8, the process of water desorption is exothermic and exergonic for the Ni-doped SnO_2_ (110) systems compared to pure SnO_2_. However, the subsequent CO_2_ adsorption on the empty site is endergonic (Fig. 8), with positive reaction-free energies for SnO_2_ (0.524 eV), Ni@SnO_2_ (0.152 eV), and 2Ni@SnO_2_ (0.194 eV) model systems which becomes more feasible if SnO_2_ is Ni doped. Further, upon abstracting an H from a co-adsorbed water molecule, *CO_2_ can form *HCOO. For Ni@SnO_2,_ the *HCOO intermediate is thermodynamically more stable (−1.374 eV, Fig. 8) compared to pure SnO_2_ (−0.594 eV, Fig. 8). Interestingly, for 2Ni@SnO_2,_ the CO_2_ molecule directly tends to form a stable HCOOH, surpassing the *HCOO intermediate state. The formation of *HCOOH from *HCOO and neighboring *OH_2_, is exergonic for SnO_2_ with a reaction-free energy of −1.021 eV, whereas for the Ni@SnO_2_ system, this process is endergonic (+0.391 eV) energy. For 2Ni@SnO_2_ the *HCOO intermediate is found to be protonated directly and form *HCOOH, an overall exergonic process with a reaction-free energy of −0.227 eV. From *HCOOH, the desorption of HCOOH is endergonic and requires 0.300 eV, 0.478 eV, and 0.610 eV for SnO_2,_ Ni@SnO_2,_ and 2Ni@SnO_2_, respectively (Fig. 8). Therefore, it is clear that under certain reaction conditions, the desorption of HCOOH can become the rate-limiting step in the reaction. Especially, for the 2Ni@SnO_2_ system, with the highest adsorption free energy barrier for *HCOOH desorption, temperature facilitates the desorption process given the decreasing free energy differences from 25 °C to 40 °C (Fig. 9). These findings agree with the temperature-dependent faradaic efficiencies and partial current densities of CO_2_ electroreduction (Fig. 5c and d). Finally, after the production of HCOOH, the catalyst needs to regenerate to continue with the catalytic cycle. The catalyst regeneration with two H^+^/e^−^ pairs is endergonic with the Δ*G* increasing in the order: 2Ni@SnO_2_ (0.097 eV) < Ni@SnO_2_ (1.092 eV) < SnO_2_ (1.308 eV), however, this regeneration can happen electrochemically, and under cathodic potentials of −0.90 V *vs.* RHE this regeneration process will be spontaneous. The regeneration process is more favorable for the surfaces with more Ni species (*e.g.*, Ni^2+^ and Ni^3+^), which indicates the influence of Ni doping on the catalytic activity of SnO_2_.

## 4. Conclusions

NiOSnO NFs have been synthesized by electrospinning. NiOSnO NFs effectively function as electrocatalysts for the electrochemical CO_2_RR, yielding HCOO^−^ beyond the room temperature suitable to current electrolyzers. The highest faradaic efficiencies to formate are achieved with NiOSnO50NF and NiOSnO75NF at an electroreduction temperature of 40 °C. XPS and EELS analyses reveal a synergistic effect between the Ni and Sn species. Electrochemical measurements and *in situ* DEMS provide insights into product distribution during CO_2_RR. Computational Pourbaix diagrams show that this synergistic effect arises from the dissolution of NiO under reducing conditions. DFT calculations show that embedding Ni in SnO_2_ is energetically more favorable in addition to aiding the reduction of the SnO_2_ surface under relevant electroreduction conditions. The desorption of HCOOH is the rate-limiting step whose free energy decreases with increasing temperature from 25 °C to 40 °C, which agrees with the temperature-dependent faradaic efficiencies and partial current densities found during the experiments. Looking into the future, it is clear that catalysts like NiOSnO NFs can be further designed for other temperature conditions rather than room temperature and will, in the future, be used in CO_2_ electrolyzer technologies over various temperature ranges. These findings underscore the significance of catalyst discovery and explore the potential for temperature-driven synergistic effects in metal oxide catalysts for CO_2_ electroreduction.

## Data availability

Data are available upon request from the authors.

## Author contributions

M. A. R. O., R. L., M. V., and A. S. A. designed the experiments, analyzed the data, and wrote the first draft of the manuscript. M. A. R. O., M. S., C. F., and E. C. -M. synthesized the material and carried out electrochemical measurements. R. L. and M. V. performed the DFT calculations (1) to construct the computational Pourbaix diagrams, and (2) to unravel the relevant CO_2_ reduction pathways. F. R. -Z. performed STEM-related analysis. The initial idea was coined by A. M., C. F., T. K., J. G. E., M. V., and A. S. A. All authors contributed to the final draft.

## Conflicts of interest

The authors declare no competing interests.

## Supplementary Material

TA-012-D4TA04116J-s001
