# Supplementary material for: Temperature promotes selectivity during electrochemical CO2 reduction on NiO:SnO2 nanofibers
Source: J Mater Chem A Mater. 2024 Aug 8;12(47):32821–35. doi: 10.1039/d4ta04116j (PMC11363033; doi:10.1039/d4ta04116j)
Supplement: TA-012-D4TA04116J-s001 [file TA-012-D4TA04116J-s001.pdf]

Supplementary Information (SI) for Journal of Materials Chemistry A.
This journal is © The Royal Society of Chemistry 2024

Temperature Promotes Selectivity During Electrochemical CO_2 Reduction on $NiO:SnO_2$ Nanofibers

M. A. Rodriguez-Olguin,[#] R. Lipin,[#] M. Suominen, F. Ruiz-Zepeda, E. Castañeda-Morales, A. Manzo-Robledo, J.G.E. Gardeniers, C. Flox,[*] T. Kallio,[*] M. Vandichel,[*] A. Susarrey-Arce[*]

[#]These authors contributed equally to this work

[*]Corresponding author(s): cristina.flox@ciiae.org; tanja.kallio@aalto.fi; matthias.vandichel@ul.ie; a.susarreyarce@utwente.nl

1. Ni- and NiO electrocatalysts

Table S1. Ni/NiO electrocatalyst used for formate and other products during CO_2RR.

Compound	Synthesis Method	Main product	Electrolyte	FE	E (vs RHE)	J_{HCOO-} (mA/cm^2)	T (°C)	Reference
**NiO@Cl-9%**	Electrospinning	$HCOO^-$	0.1M $KHCO_3$	70%	-0.8 V	14.7	25	1
**Ni-N-C**	In situ carbonization	CO	0.1 M $KHCO_3$	90%	-0.8V	5.0 (for CO)	25	2
**Ni atomic/carbon**	Pyrolysis	CO	0.5 M $KHCO_3$	90%	-1.0 V	57.1 (for CO)	25	3
**Ni single atoms/CNF**	Electrospinning	CO	0.5M $KHCO_3$	88%	-1.0 V	308.4 (for CO)	25	4
**Ni/NiO/g-C_3N_4**	Photocatalytic reduction	CO	0.5 M Na_2SO_4	87%	-0.3 V	7.8 μA/cm^2	25	5
**NiO doped Zn^{2+}**	Combustion synthesis	C_2H_4	0.5 M $NaHCO_3$	47%	-0.79 V	N.R.	25	6
**Ni-N_3**	Impregnation, ZIF formation	CO	0.5 M $KHCO_3$	95%	-1.0 V	27	25	7
**Ni-N doped CNT**	CVD	CO	0.5 KHCO3	91%	-0.74 V	29	25	8
**Ni single atoms-N-C**	Ball-milling, pyrolysis	CO	0.1 $KHCO_3$	93%	-1.3 V	16.5	25	9
**Ni-N_{4-x}-C_x**	Pyrolysis	CO	Humidified CO_2	99%	-2.0 V	470	25	10
**Co–N–Ni**	Sonochemistry	CO	0.1 M $KHCO_3$	94.6%	- 370 mV	1.7	25	11
**Ni/$Ni_3ZnC_{0.7}$-NC**	Hydrothermal	CO	0.5 $KHCO_3$	92.5%	- 0.87 V	15.77	25	12
**NiN_x/NCNT**	Ball-milling, annealing	CO	1 M KOH	99%	-0.272 V	85.6	25	13
**Ni-N_4 in mesoporus carbon**	Solvent drying and annealing	CO	0.5 $KHCO_3$	95%	-0.8 V	366	25	14
**Ni single atom/Ni NP/MOF**	Pyrolysis	CO	1 M KOH	99%	-1.82 V	160	25	15
**CuZn-Ni aerogel**	Aerogel synthesis	CO	1M KOH	80%	-0.8 V	20.0	25	16
**Ni-SAC-nano array**	Hydrothermal, annealing	CO	0.5 $KHCO_3$	90%	-1.0 V	66.0	25	17

2. Sn and SnO_2 electrocatalysts

Table S2. Sn/SnO_2 electrocatalyst for formate formation during CO_2RR.

Compound	Synthesis Method	Main product	Electrolyte	FE	E (vs RHE)	J_{HCOO-} (mA/cm^2)	T (°C)	Reference
**SnO_2-Cl doped**	Sonochemistry	$HCOO^-$	2M $KHCO_3$	59.1%	1.3	32.6	25	18
**Anodic SnO_2**	Anodic oxidation	$HCOO^-$	0.5M KHCO3	73%	0.8	10	25	19
**Mn-doped atomic SnO_2 layers**	Hydrothermal	$HCOO^-$	0.1M KHCO3	75%	1.03	21.2	25	20
**Zn-Sn oxides**	Biomineralization	$HCOO^-$	0.1M KHCO3	70%	-1.1 V	8.4 (for CO + HCOOH)	25	21
**SnO_2/C**	Impregnation	$HCOO^-$	0.5 M $KHCO_3$	92%	0.86 V	29	25	22
**Sn-halogen incorporated**	Hydrolysis	$HCOO^-$	0.5 M $KHCO_3$	96%	-0.9 V	62.4	25	23
**SnO nanosheets**	Precipitation	$HCOO^-$	1M KOH	94%	0.7V	330	25	24
**SnO_2/GO**	Hydrothermal	$HCOO^-$	0.1M $KHCO_3$	84.4%	0.96V	4.5	25	25
**VO-rich N-SnO_2**	Hydrothermal	$HCOO^-$	0.1M $KHCO_3$	83%	0.9V	10	25	26
**Ov-engineered SnO_2**	Thermal treatment	$HCOO^-$	1 M $KHCO_3$	80%	-0.9 V	16.6	25	27
**B-doped SnO_2**	Hydrothermal	$HCOO^-$	0.5M $KHCO_3$	95%	1.0 V	43.2	25	28
**SnO_2 nanosheets**	Solvothermal	$HCOO^-$	0.1M $NaHCO_3$	83%	320 mV	16	25	29
**SnO_2/OC**	Hydrothermal	$HCOO^-$	0.1M $KHCO_3$	75%	1.29 V	13.4	25	30
**SnO_x**	Deposition	$HCOO^-$	0.5M $KHCO_3$	69%	1.0	53	25	31
**Wavy SnO_2**	Hydrothermal	$HCOO^-$	0.5M $KHCO_3$	22%	1.0 V	87	25	32
**Porous SnO_2 nanosheets**	CVD	$HCOO^-$	0.5M $KHCO_3$	94%	0.51 V	18.8	25	33
**Chainlike Mesoporous SnO_2**	Anodic oxidation	$HCOO^-$	0.1M $KHCO_3$	82%	1.06 V	15.3	25	34
**SnO_2-GQDs**	Hydrothermal	$HCOO^-$	0.1M $KHCO_3$	93%	1.3V	16.2	25	35
**SnO_x Nanosheets-MWCNTs**	Hydrothermal	$HCOO^-$	0.5M $KHCO_3$	77%	1.25V (vs SHE)	11.0	25	36
**Double-shell SnO_x nanosphere**	Hydrothermal	$HCOO^-$	0.5M $KHCO_3$	81%	1.15 V	44.7	25	37
**1D SnO_2**	Electrospinning	$HCOO^-$	0.1M $KHCO_3$	70%	1.29 V	12.0	25	38
**Sn/SnO_2 nanofiber**	Electrospinning	$HCOO^-$	0.1M $KHCO_3$	82.1%	1.6 V (vs SCE)	22.9	25	39

3. Ni:Sn metal or metal oxide electrocatalysts

Table S3. Ni:Sn metal or metal oxide electrocatalyst used for formate formation during CO_2RR.

Compound	Synthesis Method	Main product	Electrolyte	FE	E (vs RHE)	J_{HCOO-} (mA/cm^2)	T (^oC)	Reference
**N_4-Ni-Sn-N_4 SAC**	Impregnation	HCOOH	0.5 M $KHCO_3$	86.1%	-0.82 V	43.7	25	[40]
**Ni-doped SnO_2**	Solvothermal	HCOOH	1 M KOH	80%	-1.2 V	116	25	[41]

4. SnO_2-based model

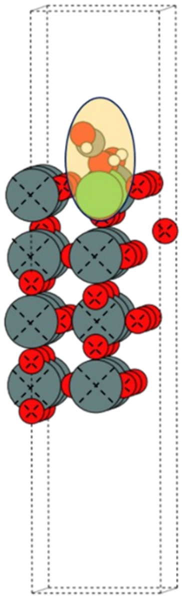

Figure S1. An ASE-GUI representation of the model system for the vibrational analysis. The constrained atoms are marked with a dashed X, and the unfixed atoms are highlighted in yellow.[42]

Table S4. Thermodynamic quantities for the gas phase molecules in eV.

	E_{DFT}	ZPE	$\int_0^{298} C_v dT$	TS	G
H_2	-6.7619	0.27	0.09	0.43	-6.832
H_2O	-14.2336	0.56	0.10	0.67	-14.24
CO_2	-23.0229	0.31	0.10	0.66	-23.273
HCOOH	-29.8814	0.89	0.11	1.05	-29.93

5. Morphological inspection for NiO, SnO_2, and NiOSnO25NF

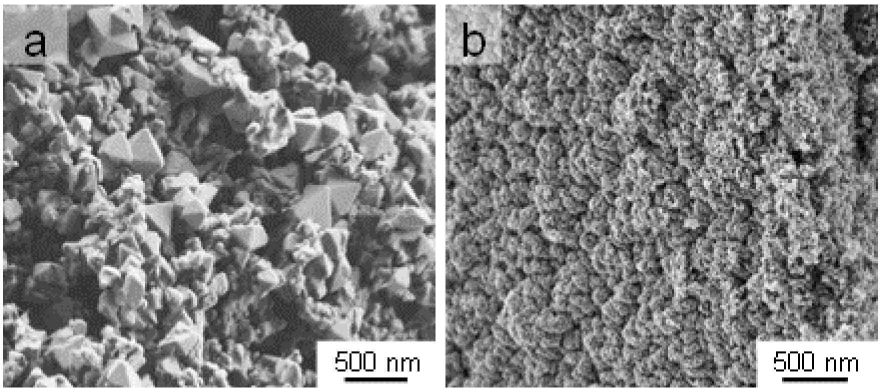

Figure S2. SEM image of (a) NiO and (b) SnO_2.

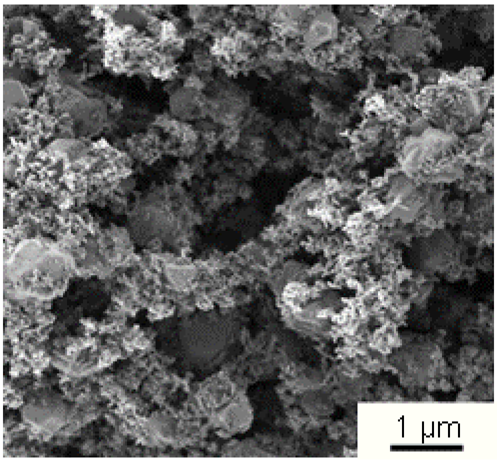

Figure S3. SEM image of NiOSnO25NF.

6. Interface structure between NiO and SnO_2 nanocrystals

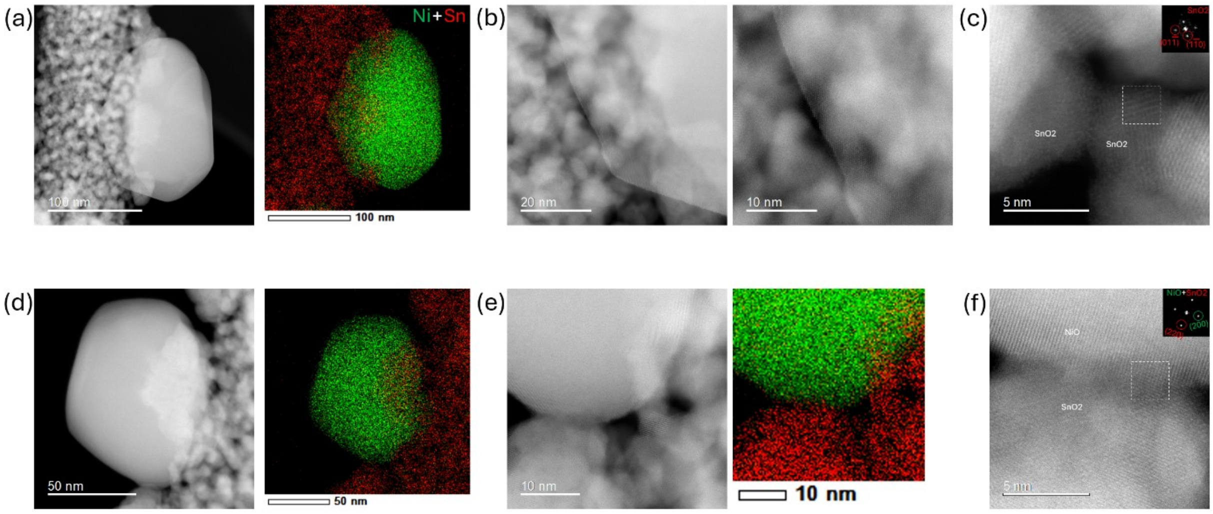

Figure S4. Interface structure between NiO and SnO2 nanocrystallites. (a) ADF and EDX map of the NiO crystallite decorating a nanofiber. (b) Close-up of a NiO crystallite edge laying on the nanofiber with multiple SnO_2 nanocrystallites homogeneously assembled. (c) An interface between two SnO_2 nanocrystallites shows that the contact planes for one of the nanocrystallites observed along the zone axis [111] correspond to 011. (d) ADF and EDX map of a second example of a NiO crystallite sitting on the nanofiber. (e) A close-up ADF and EDX map showing the interaction on multiple contact points of the large NiO crystallite with the SnO_2 nanocrystallites. (f) Interface between the NiO crystallite and SnO_2 nanocrystallites. The exposed facets of the observed SnO_2 nanocrystallites correspond to 220 planes, while the NiO crystallite facet in contact exhibits a surface with multiple steps that are oriented perpendicular to 200 planes. This illustrates that the NiO crystallite fits a non-sharp interface but is still aligned with the multigrain arrangement of the SnO_2 nanoparticles that construct the nanofiber.

7. Electrochemical impedance spectroscopy

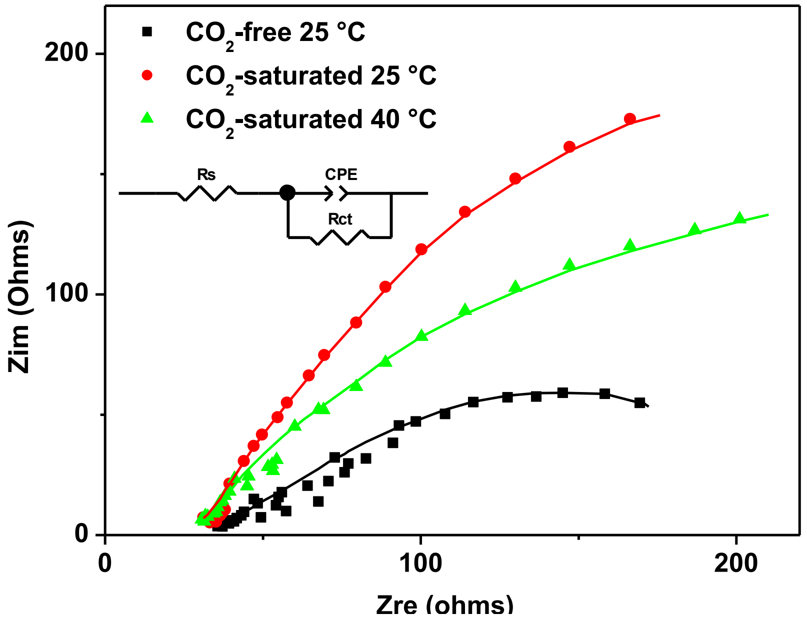

Figure S5. Nyquist plots for NiOSnO75NF without and saturated with CO_2 at different temperatures in 0.1 M $KHCO_3$

Table S5. Electric parameters for NiOSnO75NF over various temperatures. For the experimental results, see **Figure S5**.

**Conditions**	**Rs**	**Rct**	**CPE-P**	**CPE-T**
CO_2-free (25 °C)	33.16	424.60	0.45191	0.00451
CO_2-saturated (25 °C)	31.12	764.51	0.65253	0.00291
CO_2-saturated (40 °C)	33.74	511.75	0.59552	0.00395

The Rct values in **Table S5** indicate that NiOSnO75NF has a higher affinity to CO_2. In the absence of CO_2, the affinity to H_2 might be preferred.

8. Linear scan voltammetry

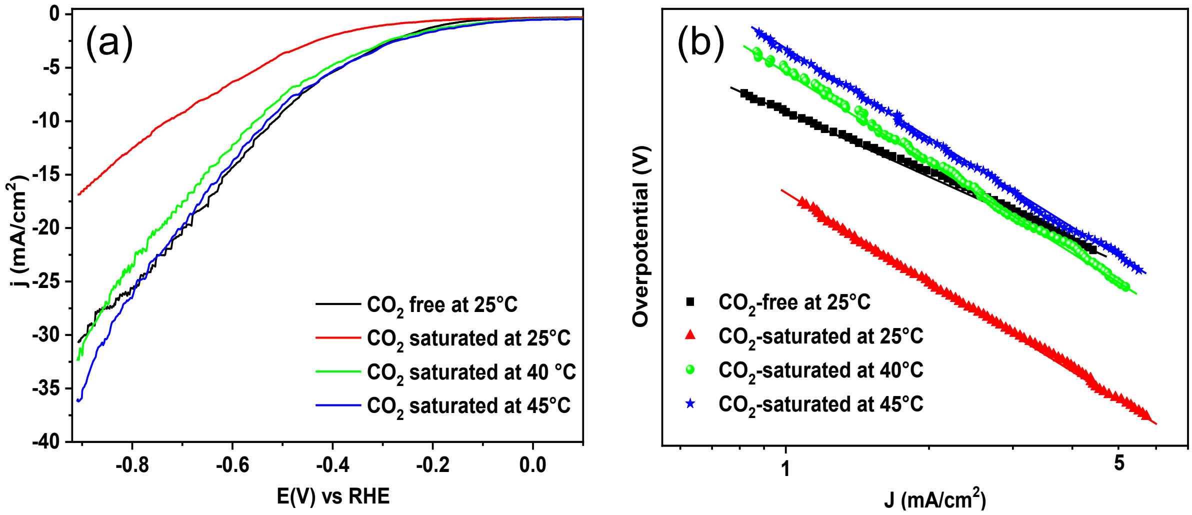

Figure S6. (a) Linear scan voltammetry (LSV) and (b) Tafel slopes derived from LSV in the absence and presence of CO_2 at 25 °C (black line/closed square circles and red line/closed triangles), 40 °C (green line/closed green circles), and 45°C (blue line/blue asterisks) for NiOSnO75NF in 0.1 M $KHCO_3$.

Table S6. Tafel slopes for NiOSnO75NF over various temperatures. For the experimental results, see **Figure S6**.

**Conditions**	**Tafel Slope (mV/dec)**
CO_2-free (25 °C)	210
CO_2-saturated (25 °C)	302
CO_2-saturated (40 °C)	293
CO_2-saturated (45 °C)	278

9. Electrochemical measurements and HCOOH fragments

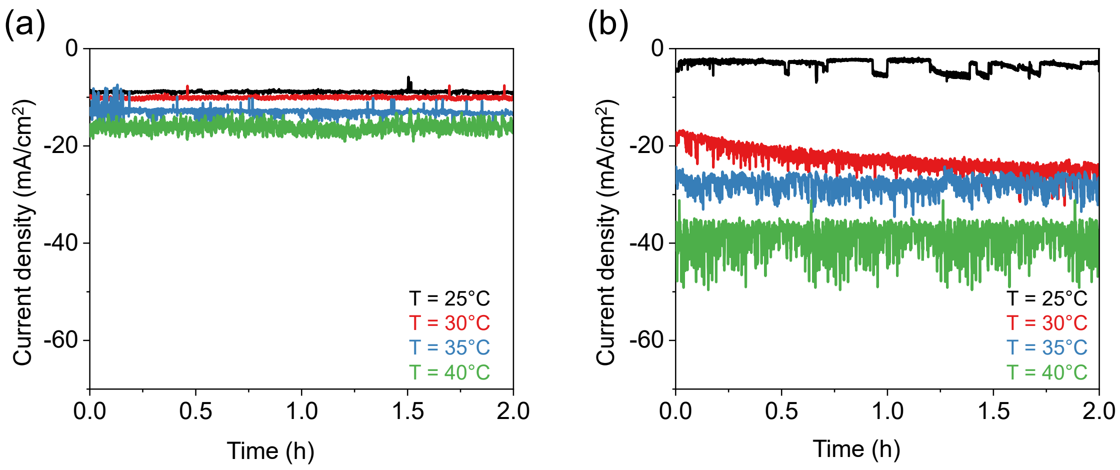

Figure S7. Chronoamperogram of NiOSn50NF and NiOSnO75NF in the presence of CO_2 over various temperatures and applied cell potentials of -0.85 vs. RHE.

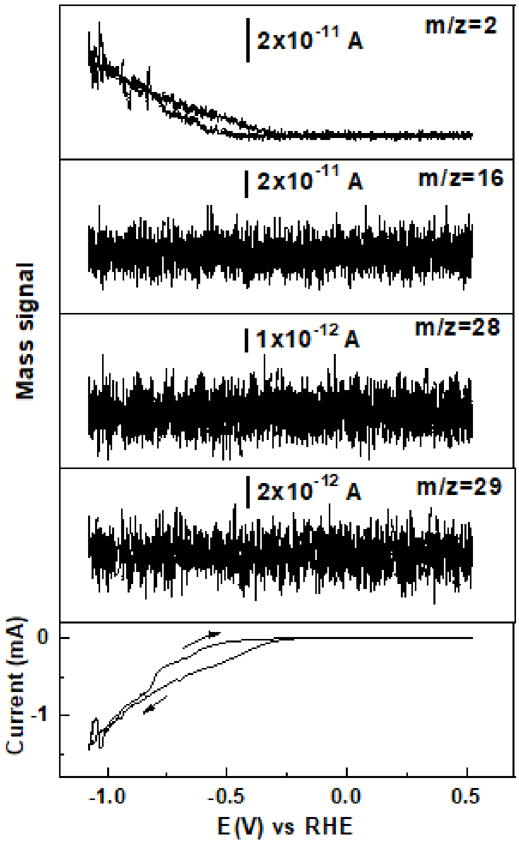

Figure S8. CV characteristic for NiOSnO75NF at 25 °C in the absence of CO_2. The mass signal is also shown as a function of the applied potential (1 mV/s) for m/z = 2, m/z = 16, m/z = 28, and m/z = 29.

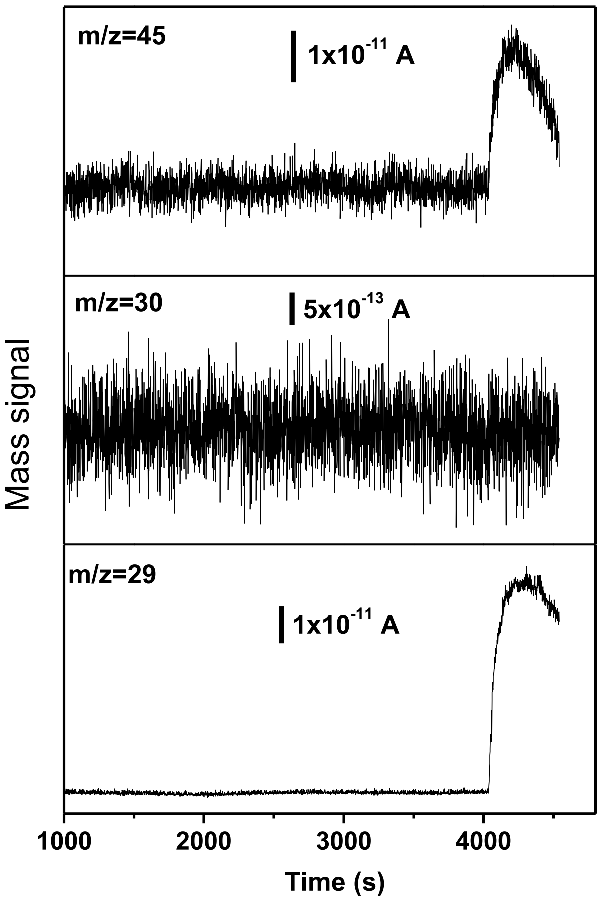

Figure S9. The mass signal of HCOOH for m/z = 45, m/z = 30, and m/z = 29 fragments.

10. Chemical characterization with STEM-EDX

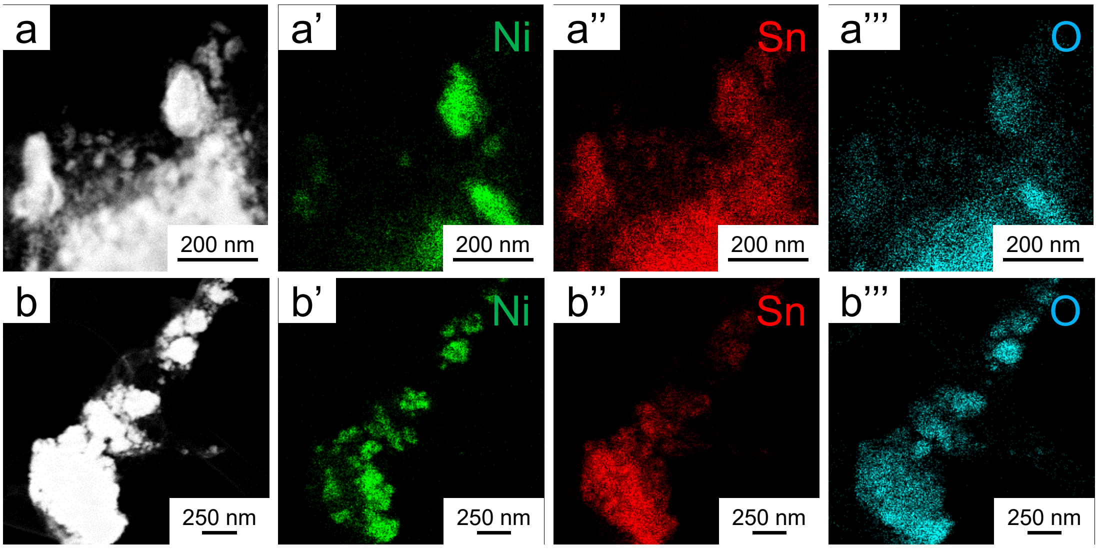

Figure S10. Representative STEM-ADF and STEM-EDX maps for NiOSnO75NF after 2 h (a) and 22 h (b) of CO_2 electrolysis.

11. Raman

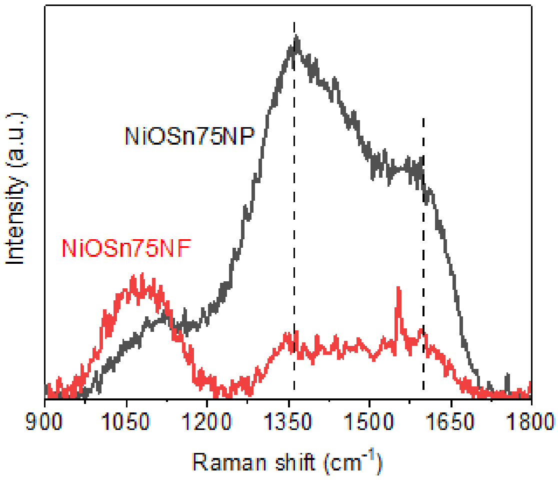

Figure S11. Representative Raman spectra for NiOSnO75NF and NiOSnO75NP

In **Figure S11**, the Raman spectrum is shown. The first peak at 1359 cm^{-1} is identified as a defective or disordered carbon lattice.[43] The peak at 1596 cm^{-1} correlates with graphitic carbon species.[44,45] The results demonstrate that NiOSnO75NP retains more carbon remnants than NiOSnO75NF.

12. STEM-ADF micrographs for NiOSnO75NF with and without surfactant

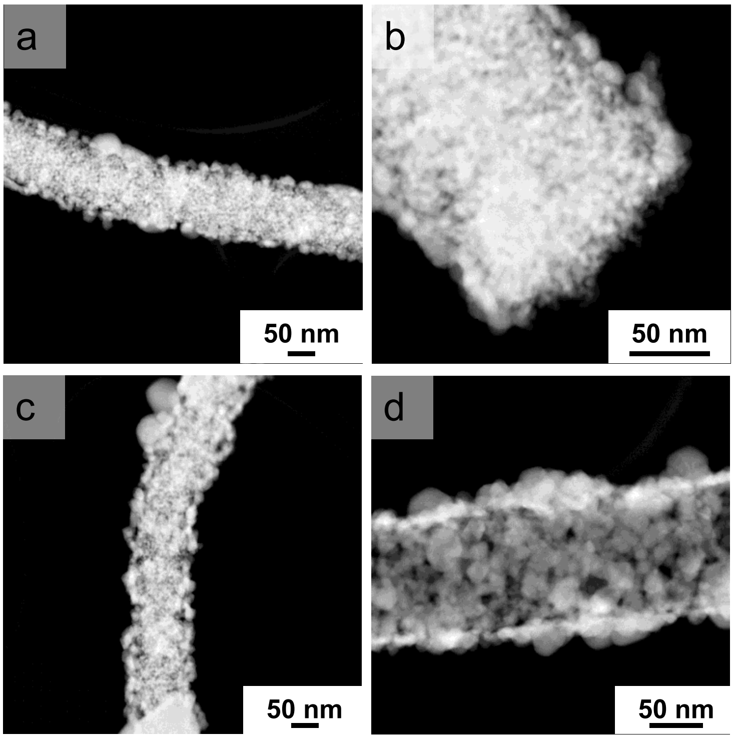

Figure S12. STEM-ADF micrographs of (a-b) NiOSnO75NF and (c-d) NiOSnO75 with surfactant.

13. CO_2RR for NiOSnO75NF with surfactant

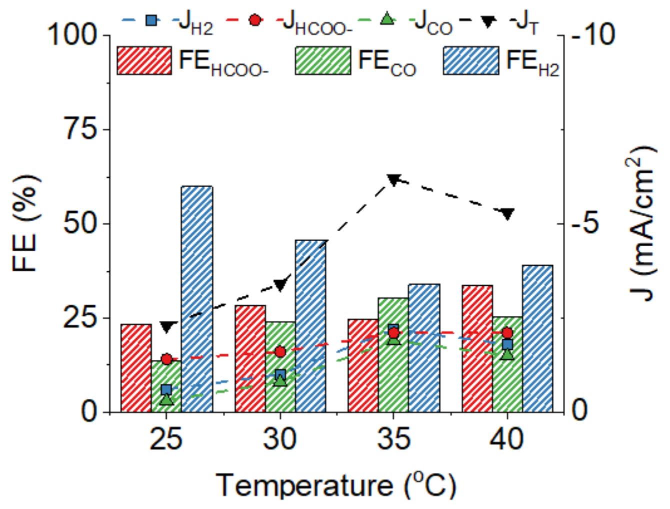

Figure S13. Product distribution for NiOSnO75NF with surfactant at -0.85 vs. RHE for 2 h over various temperatures, i.e., 25, 30, 35, 40 °C.

14. Computational Insights

Cohesive Energy: Cohesive energy is considered a well-known descriptor to estimate the stability of surface slabs for bulk structures.[46] The relative stabilities of Ni-doped SnO_2 are examined from the SnO_2 and NiO bulk structures. The cohesive energy ($\mathbf{E_{coh}}$) for an undoped SnO_2(hkl) slab can be expressed as follows:

$$E_{Coh} = \frac{E_{SnO_2(hkl)} - (N_{units}^{SnO_2} E_{bulk}^{SnO_2})}{(a+b)} \quad (1)$$

where $E_{SnO_2(hkl)}$, $E_{bulk}^{SnO_2}$ are the energies of a pristine SnO_2 slab and bulk SnO_2 in a tetragonal lattice, respectively. $N_{units}^{SnO_2}$, a and b are the number of SnO_2 per unit formula, Sn and O atoms, respectively. Furthermore, the cohesive energy for Ni-doped SnO_2 systems can be computationally determined:

$$E_{Coh} = \frac{E_{Ni@SnO_2(hkl)} - (N_{units}^{SnO_2} E_{bulk}^{SnO_2} + N_{units}^{NiO} E_{bulk}^{NiO}) + 0.5 N_{O_2} E_{O_2}}{(a+b+c)} \quad (2)$$

where $E_{Ni@SnO_2(hkl)}$, is the total energy of the doped SnO_2 surface slab, and E_{bulk}^{NiO}, E_{O_2} are the energies of bulk NiO in a cubic lattice and O_2 gas molecule, respectively. The factors a, b, and c are the number of Sn, Ni, and O in the Ni-doped systems. The more negative the cohesive energy is compared to pure SnO_2, the more stable the doped phase.[47] The cohesive energy per atom increases in the order 2Ni@SnO_2 > Ni@SnO_2 > SnO_2 (**Table S7**). As the concentration of the Ni increases, the cohesive energy becomes more negative, implying the desirable formation of Ni-doped phases.

Surface Formation Energy: The surface formation energy (γ) is the energy needed to create a surface with (hkl) termination from its bulk and is often used as a descriptor for surface stability.[48,49] Herein, model systems with a (110) termination of SnO_2 are studied because it is the most abundant surface in all experimental samples studied, as identified by experimental XRD data (**Figure 3**). A surface with low γ implies a stable surface. The surface formation energy for the (110) terminated SnO_2 is given as:

$$\gamma_{\boldsymbol{SnO_2}(\mathbf{110})} = \frac{1}{2A}(E_{SnO_2} - N_{units}^{SnO_2} E_{bulk}^{SnO_2}) \quad (3)$$

Here, A represents the surface area multiplied with a factor 2 because of the assumption of symmetric terminations of the $SnO_2(110)$ model. Note that for the $SnO_2(110)$, there are several possible terminations, and herein, we considered the O-terminated surface as it has garnered significant interest in previous theoretical investigations.[50–52] However, in the case of Ni doping, the terminations will become asymmetric, and the surface formation energy of the Ni-doped side of the model system can then be defined as:

$$\gamma = \gamma_{\boldsymbol{Ni@SnO_2}(\mathbf{110})} - \gamma_{\boldsymbol{SnO_2}(\mathbf{110})} \qquad (4)$$

where $\gamma_{Ni@SnO_2(110)}$ and $\gamma_{SnO_2(110)}$ are the surface formation energies of the $Ni@SnO_2$ and pristine SnO_2 model systems (in **Table S2** and **Figure S11-13**, γ or surface formation energy of the modified surface is displayed). $\gamma_{Ni@SnO_2(110)}$ is calculated as:

$$\gamma_{\boldsymbol{Ni@SnO_2}(\mathbf{110})} = \frac{1}{\mathrm{A}}(\mathrm{E}_{\mathrm{Ni@SnO_2(110)}} - (\mathrm{N}_{\mathrm{units}}^{\mathrm{SnO_2}}\mathrm{E}_{\mathrm{bulk}}^{\mathrm{SnO_2}} + \mathrm{N}_{\mathrm{units}}^{\mathrm{NiO}}\mathrm{E}_{\mathrm{bulk}}^{\mathrm{NiO}}) + 0.5\mathrm{N}_{\mathrm{O_2}}\mathrm{E}_{\mathrm{O_2}}) \qquad (5)$$

Table S7 shows the surface formation energies of Ni-doped and pristine SnO_2 systems. Like the cohesive energy, the surface energies of the Ni-doped system are low (and negative) compared to SnO_2, representing a stable surface for the doped systems. Overall, the cohesive and surface energies from the bulk oxides and O_2 confirm the higher stability of the Ni-doped $SnO_2(110)$ phases over pristine $SnO_2(110)$ phases.

Table S7. Cohesive (in eV/atom) and surface formation energies (in eV/Å^2**)** of the slabs studied.

	**E_{coh} (in eV/atom)**	**Surface Formation Energy (eV/Å^2)**
**SnO_2(110)**	0.16	0.09
**Ni@SnO_2(110)**	0.06	-0.02
**2Ni@SnO_2(110)**	-0.04	-0.21

15. Computational Insights on NiO/SnO_2 Interface

The stability of the interface between the NiO and SnO_2 is investigated by constructing a large 3×2 supercell of the existing SnO_2(110) model with four SnO_2 layers as previously used. Due to the complexity of the large supercell models (~450 atoms), we performed non-spin polarized calculations with the Brillouin zone sampling restricted to the Γ-point, keeping all other parameters unchanged. The surface formation energy (SFE) of various Ni-containing surfaces on both pristine and reduced SnO_2 (SnO/SnO_2) are calculated using equation (4) and (5). It should be noted that the surface Pourbaix diagrams in **Figure 6** indicate that the surface tends to reduce under experimental conditions, making it crucial to consider reduced SnO_2 surface models when describing NiO/SnO_2 interfaces.

The different Ni-containing systems considered are: (1) Ni at adatom positions, (2) Ni doped at six-coordinated Sn surface positions (position 1), and (3) Ni doped at five-coordinated Sn surface positions (position 2). We have also constructed a NiO ($Ni_{32}O_{32}$) nanocube adsorbed and a system with all surface Sn atoms replaced by Ni atoms (NiO/SnO_2 and NiO_2/SnO_2).

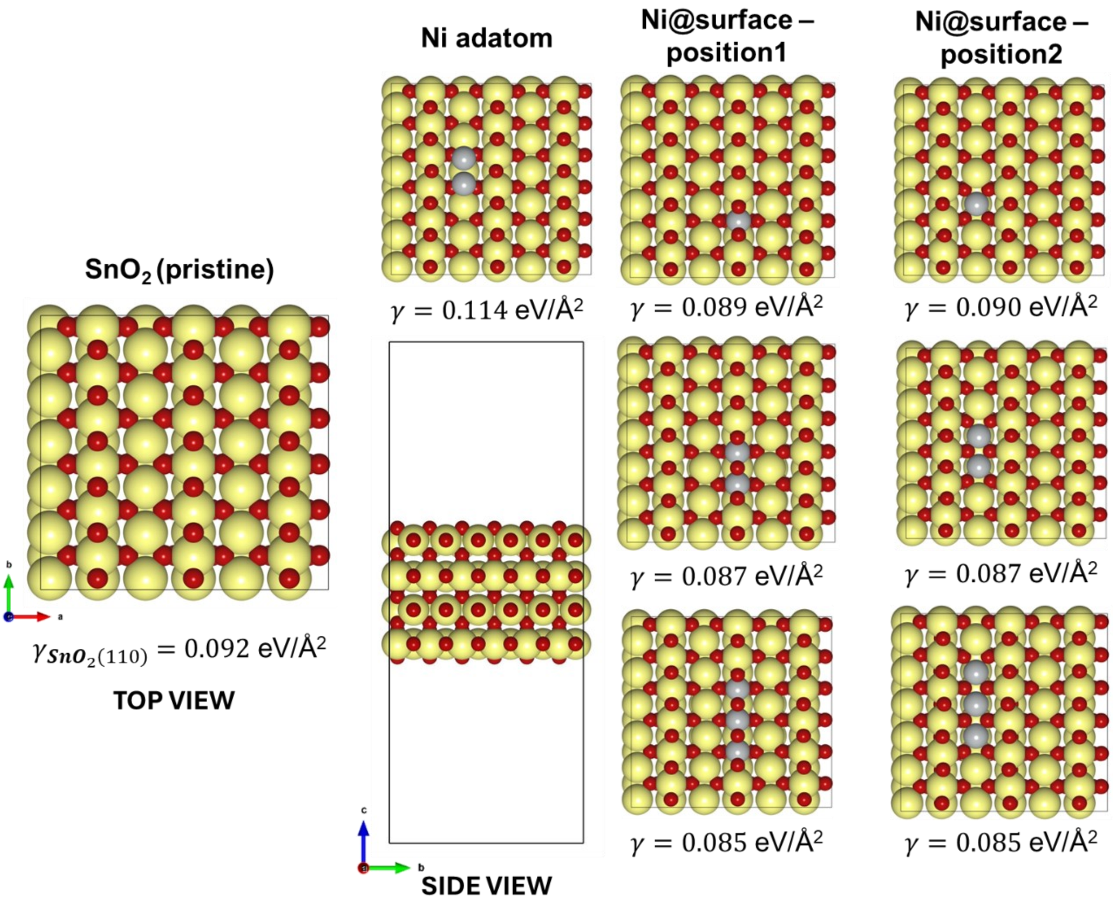

--*

Figure S14. Optimized geometries of different Ni-containing systems over SnO_2 and their corresponding surface formation energies in eV/Å^2 calculated using Eq. 4. Color codes: Sn (yellow), O (red), and Ni (grey).

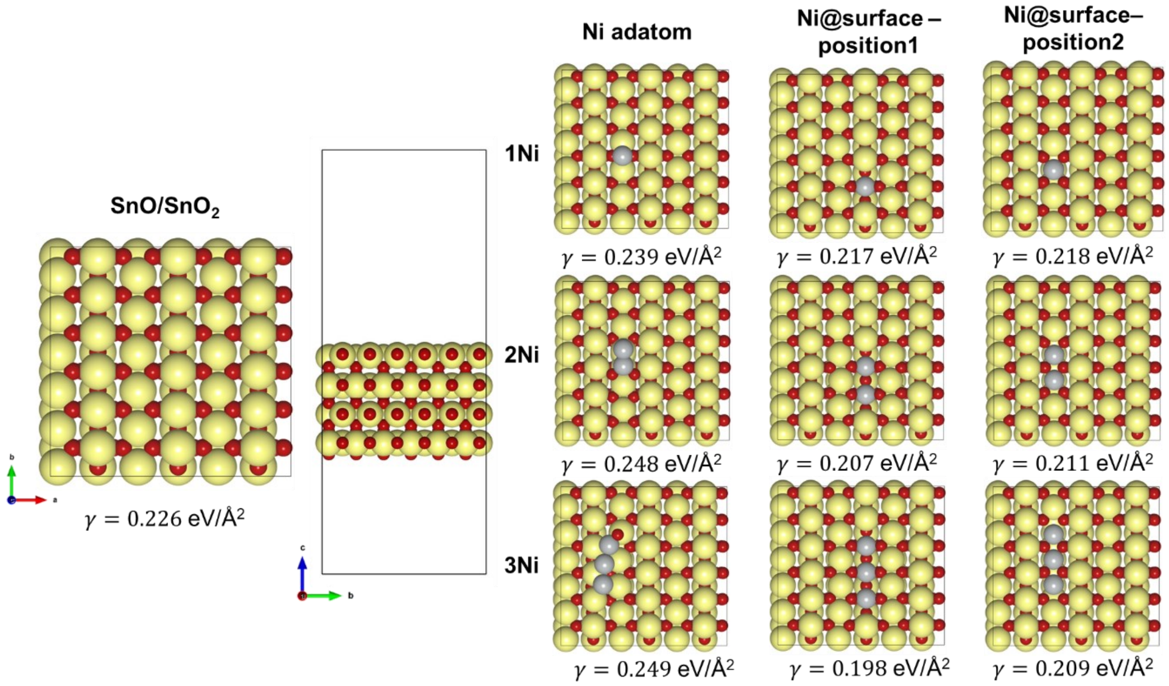

Figure S15. Optimized geometries of different Ni-containing systems over reduced SnO_2 (SnO/SnO_2) and their corresponding surface formation energies in eV/Å^2 calculated using Eq. 4. Color codes: Sn (yellow), O (red), and Ni (grey).

From **Figure S14 and S15**, it is observed that Ni lowers the surface formation energy with respect to pristine SnO_2, where the Ni atoms pull and compress the surrounding atoms from their lattice positions, similar to the small models discussed previously. The surface formation energy of 1.00 ML NiO overlayer on a reduced surface (NiO /SnO_2) was the lowest observed, with –0.347 eV/Å^2 compared to the various Ni-containing systems studied (**Figure S16**). The same configuration on the pristine SnO_2 (NiO_2/SnO_2) also exhibited a lower surface energy (0.049 eV/Å^2), the least among the Ni-containing systems with pristine SnO_2. Overall, the formation energies are an indication of the plausibility for a NiO phase supported by SnO_2 under the electrochemical conditions, corroborating experimental observations (**Figure S4**).

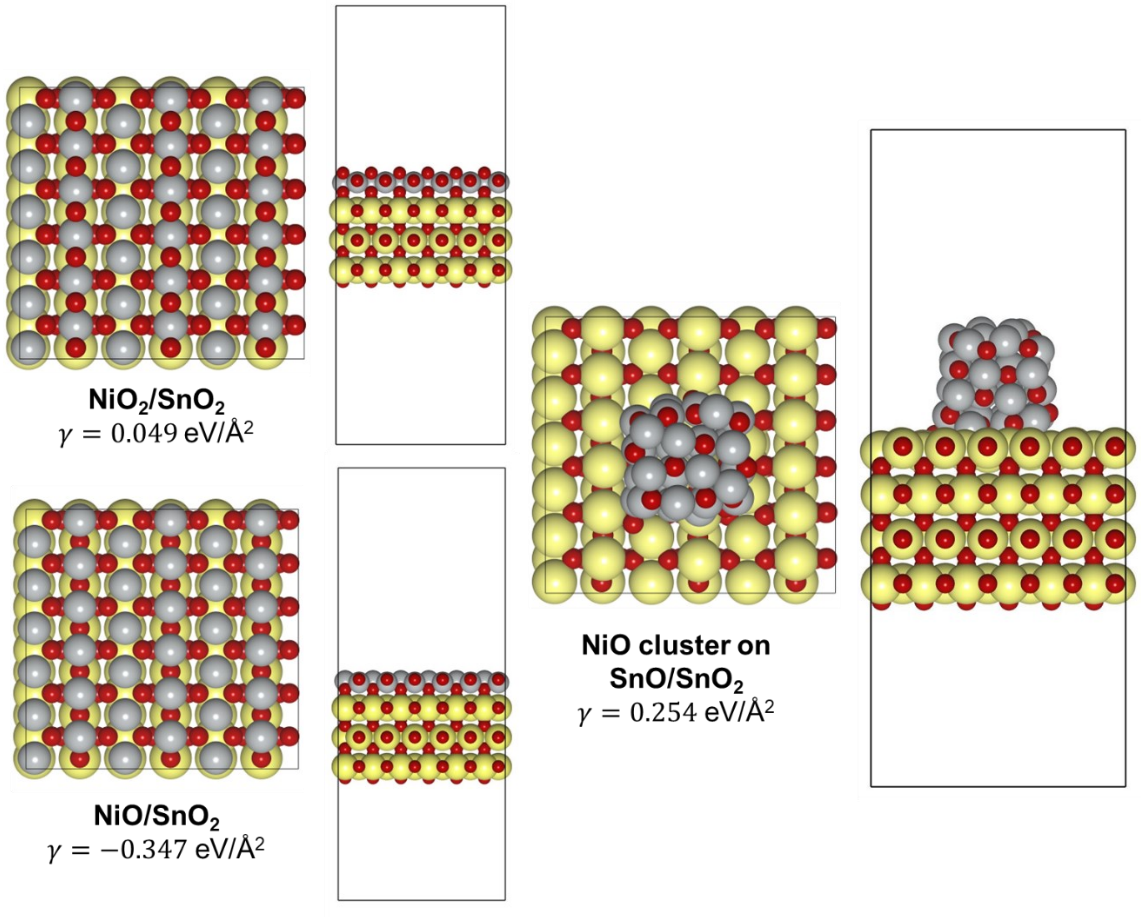

Figure S16. Optimized geometries of different NiO systems over pristine and reduced SnO_2 (SnO/SnO_2) models and their corresponding surface formation energies in eV/Å^2 calculated using Eq. 4. Color codes: Sn (yellow), O (red), and Ni (grey).

16. Gibbs Free Energy Vs Potential (pH=0)

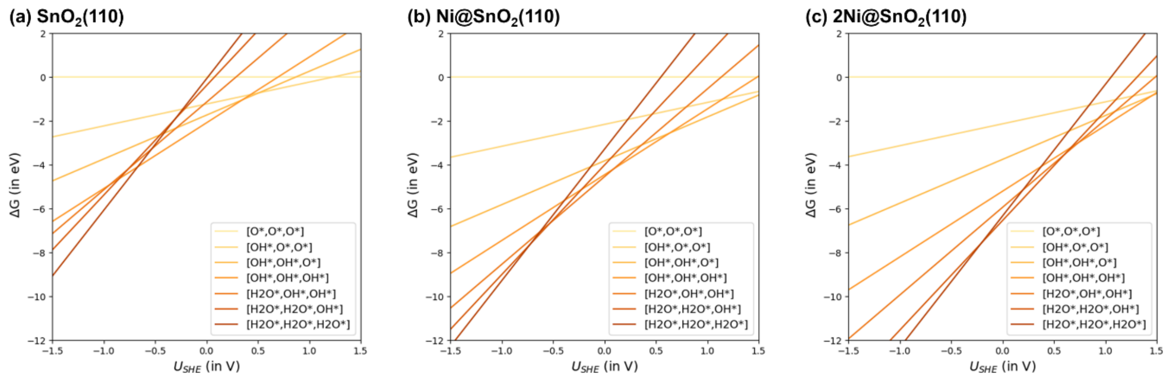

Figure S17. Gibbs free energy profiles as a function of U_{SHE} diagrams at pH = 0 for the studied $SnO_2(110)$ models.

References

1 M. A. Rodriguez-Olguin, C. Flox, R. Ponce-Pérez, R. Lipin, F. Ruiz-Zepeda, J. P. Winczewski, T. Kallio, M. Vandichel, J. Guerrero-Sánchez, J. G. E. Gardeniers, N. Takeuchi and A. Susarrey-Arce, *Appl Mater Today*, 2022, **28**, 101528.

2 Z. L. Wang, J. Choi, M. Xu, X. Hao, H. Zhang, Z. Jiang, M. Zuo, J. Kim, W. Zhou, X. Meng, Q. Yu, Z. Sun, S. Wei, J. Ye, G. G. Wallace, D. L. Officer and Y. Yamauchi, *ChemSusChem*, 2020, **13**, 929–937.

3 P. Lu, Y. Yang, J. Yao, M. Wang, S. Dipazir, M. Yuan, J. Zhang, X. Wang, Z. Xie and G. Zhang, *Appl Catal B*, 2019, **241**, 113–119.

4 H. Yang, Q. Lin, C. Zhang, X. Yu, Z. Cheng, G. Li, Q. Hu, X. Ren, Q. Zhang, J. Liu and C. He, *Nat Commun*, 2020, **11**, 593.

5 C. Han, R. Zhang, Y. Ye, L. Wang, Z. Ma, F. Su, H. Xie, Y. Zhou, P. K. Wong and L. Ye, *J Mater Chem A Mater*, 2019, **7**, 9726–9735.

6 S. Nellaiappan and S. Sharma, *ACS Appl Energy Mater*, 2019, **2**, 2998–3003.

7 Q. X. Li, D. H. Si, W. Lin, Y. B. Wang, H. J. Zhu, Y. B. Huang and R. Cao, *Sci China Chem*, 2022, **65**, 1584–1593.

8 Y. Gang, E. Sarnello, J. Pellessier, S. Fang, M. Suarez, F. Pan, Z. Du, P. Zhang, L. Fang, Y. Liu, T. Li, H. C. Zhou, Y. H. Hu and Y. Li, *ACS Catal*, 2021, **11**, 10333–10344.

9 H. Li, K. Gan, R. Li, H. Huang, J. Niu, Z. Chen, J. Zhou, Y. Yu, J. Qiu and X. He, *Adv Funct Mater*, 2023, **33**, 2208622.

10 J. Leverett, J. A. Yuwono, P. Kumar, T. Tran-Phu, J. Qu, J. Cairney, X. Wang, A. N. Simonov, R. K. Hocking, B. Johannessen, L. Dai, R. Daiyan and R. Amal, *ACS Energy Lett*, 2022, **7**, 920–928.

11 J. Pei, T. Wang, R. Sui, X. Zhang, D. Zhou, F. Qin, X. Zhao, Q. Liu, W. Yan, J. Dong, L. Zheng, A. Li, J. Mao, W. Zhu, W. Chen and Z. Zhuang, *Energy Environ Sci*, 2021, **14**, 3019–3028.

12 L. Liao, C. Jia, S. Wu, S. Yu, Z. Wen and S. Ci, *Nanoscale*, 2024, **16**, 8119–8131.

13 R. Zhao, Y. Wang, G. Ji, J. Zhong, F. Zhang, M. Chen, S. Tong, P. Wang, Z. Wu, B. Han and Z. Liu, *Advanced Materials*, 2023, **35**, 2205262.

14 B. Chen, B. Li, Z. Tian, W. Liu, W. P. Liu, W. Sun, K. Wang, L. Chen and J. Jiang, *Adv Energy Mater*, 2021, **11**, 2102152.

15 C. F. Wen, F. Mao, Y. Liu, X. Y. Zhang, H. Q. Fu, L. R. Zheng, P. F. Liu and H. G. Yang, *ACS Catal*, 2020, **10**, 1086–1093.

16 J. Chen, X. Wei, R. Cai, J. Ren, M. Ju, X. Lu, X. Long and S. Yang, *ACS Mater Lett*, 2022, **4**, 497–504.

17 Z. Ma, T. Zhang, L. Lin, A. Han and J. Liu, *AIChE Journal*, 2023, **69**, e18161.

18 D. Sassone, J. Zeng, M. Fontana, M. A. Farkhondehfal, C. F. Pirri and S. Bocchini, *ACS Appl Mater Interfaces*, 2022, **14**, 42144–42152.

19 R. Ma, Y. L. Chen, Y. Shen, H. Wang, W. Zhang, S. S. Pang, J. Huang, Y. Han and Y. Zhao, *RSC Adv*, 2020, **10**, 22828–22835.

20 Y. Wei, J. Liu, F. Cheng and J. Chen, *J Mater Chem A*, 2019, **7**, 19651–19656.

21 J. Jiang, B. Huang, R. Daiyan, B. Subhash, C. Tsounis, Z. Ma, C. Han, Y. Zhao, L. H. Effendi, L. C. Gallington, J. N. Hart, J. A. Scott and N. M. Bedford, *Nano Energy*, 2022, **101**, 107593.

22 Y. He, W. J. Jiang, Y. Zhang, L. B. Huang and J. S. Hu, *J Mater Chem A*, 2019, **7**, 18428–18433.

23 T. Wang, J. Chen, X. Ren, J. Zhang, J. Ding, Y. Liu, K. H. Lim, J. Wang, X. Li, H. Yang, Y. Huang, S. Kawi and B. Liu, *Angewandte Chemie*, 2023, **62**, e202211174.

24 Y. Qian, Y. Liu, H. Tang and B. L. Lin, *J CO2 Util*, 2020, **42**, 101287.

25 Z. Yang, C. Yang, J. Han, W. Zhao, S. Shao, S. Li, H. Gao, H. Xie and X. Zhang, *J Mater Chem A*, 2021, **9**, 19681–19686.

26 Z. Li, A. Cao, Q. Zheng, Y. Fu, T. Wang, K. T. Arul, J. L. Chen, B. Yang, N. M. Adli, L. Lei, C. L. Dong, J. Xiao, G. Wu and Y. Hou, *Adv Mater*, 2021, **33**, 2005113.

27 B. Ning, M. Liu, Y. Hu, H. Jiang and C. Li, *Dalton Transactions*, 2022, **51**, 3512–3519.

28 X. Zhong, T. Yang, S. Liang, Z. Zhong and H. Deng, *Small*, 2023, **19**, 2303185.

29 N. Han, Y. Wang, J. Deng, J. Zhou, Y. Wu, H. Yang, P. Ding and Y. Li, *J Mater Chem A*, 2019, **7**, 1267–1272.

30 Z. Kuang, W. Zhao, C. Peng, Q. Zhang, Y. Xue, Z. Li, H. Yao, X. Zhou and H. Chen, *ChemSusChem*, 2020, **13**, 5896–5900.

31 L. G. Puppin, L. F. da Silva, M. Carmo, H. Varela and O. F. Lopes, *J Mater Res*, 2021, **36**, 4240–4248.

32 Z. Chen, T. Fan, Y. Q. Zhang, J. Xiao, M. Gao, N. Duan, J. Zhang, J. Li, Q. Liu, X. Yi and J. L. Luo, *Appl Catal B*, 2020, **261**, 118243.

33 G. Liu, Z. Li, J. Shi, K. Sun, Y. Ji, Z. Wang, Y. Qiu, Y. Liu, Z. Wang and P. A. Hu, *Appl Catal B*, 2020, **260**, 118134.

34 K. Bejtka, J. Zeng, A. Sacco, M. Castellino, S. Hernández, M. A. Farkhondehfal, U. Savino, S. Ansaloni, C. F. Pirri and A. Chiodoni, *ACS Appl Energy Mater*, 2019, **2**, 3081–3091.

35 Z. Wang, W. Tian, J. Zhan, Y. You, L. H. Zhang and F. Yu, *Ind Eng Chem Res*, 2023, **62**, 4940–4946.

36 Q. Zhang, Y. Zhang, J. Mao, J. Liu, Y. Zhou, D. Guay and J. Qiao, *ChemSusChem*, 2019, **12**, 1443–1450.

37 K. Xu, S. Liu, Z. Cao, Y. Mao and Q. Mao, *Electrochem commun*, 2021, **128**, 107056.

38 L. Fan, Z. Xia, M. Xu, Y. Lu and Z. Li, *Adv Funct Mater*, 2018, **28**, 1706289.

39 H. Hu, L. Gui, W. Zhou, J. Sun, J. Xu, Q. Wang, B. He and L. Zhao, *Electrochim Acta*, 2018, **285**, 70–77.

40 W. Xie, H. Li, G. Cui, J. Li, Y. Song, S. Li, X. Zhang, J. Y. Lee, M. Shao and M. Wei, *Angewandte Chemie*, 2021, **133**, 7458–7464.

41 M. S. Amer, H. A. AlOraij and A. M. Al-Mayouf, *J CO2 Util*, 2024, **82**, 102742.

42 A. Hjorth Larsen, J. Jørgen Mortensen, J. Blomqvist, I. E. Castelli, R. Christensen, M. Dułak, J. Friis, M. N. Groves, B. Hammer, C. Hargus, E. D. Hermes, P. C. Jennings, P. Bjerre Jensen, J. Kermode, J. R. Kitchin, E. Leonhard Kolsbjerg, J. Kubal, K. Kaasbjerg, S. Lysgaard, J. Bergmann Maronsson, T. Maxson, T. Olsen, L. Pastewka, A. Peterson, C. Rostgaard, J. Schiøtz, O. Schütt, M. Strange, K. S. Thygesen, T. Vegge, L. Vilhelmsen, M. Walter, Z. Zeng and K. W. Jacobsen, *Journal of Physics: Condensed Matter*, 2017, **29**, 273002.

43 N. A. Solopova, N. Dubrovinskaia and L. Dubrovinsky, *Appl Phys Lett*, 2013, **102**, 1–5.

44 A. Zakhurdaeva, P. I. Dietrich, H. Hölscher, C. Koos, J. G. Korvink and S. Sharma, *Micromachines (Basel)*, 2017, **8**, 1–10.

45 S. K. Jerng, D. S. Yu, J. H. Lee, C. Kim, S. Yoon and S. H. Chun, *Nanoscale Res Lett*, 2011, **6**, 1–6.

46 Q. Zhang, H. Zhang and X. L. Cheng, *Chin Physics B*, 2018, **27**, 027301.

47 J. Su, Y. Pei, Z. Yang and X. Wang, *RSC Adv*, 2015, **5**, 27229–27234.

48 Y. H. Zhao, H. Y. Su, K. Sun, J. Liu and W. X. Li, *Surf Sci*, 2012, **606**, 598–604.

49 E. Rugut, D. Joubert and G. Jones, *Comput Mater Sci*, 2021, **187**, 110099.

50 L. Braglia, M. Fracchia, P. Ghigna, A. Minguzzi, D. Meroni, R. Edla, M. Vandichel, E. Ahlberg, G. Cerrato and P. Torelli, *J Phys Chem C*, 2020, **124**, 14202–14212.

51 M. Calatayud, J. Andrés and A. Beltrán, *Surf Sci*, 1999, **430**, 213–222.

52 X. Wang, H. Qin, Y. Chen and J. Hu, *J Phys Chem C*, 2014, **118**, 28548–28561.
